# Diagnostic accuracy of neonatal foot length to identify preterm and low birthweight infants: a systematic review and meta-analysis

**DOI:** 10.1136/bmjgh-2020-002976

**Published:** 2020-11-18

**Authors:** Lian V Folger, Pratik Panchal, Michelle Eglovitch, Rachel Whelan, Anne CC Lee

**Affiliations:** 1 Department of Pediatric Newborn Medicine; Global Advancement of Infants and Mothers (AIM) Lab, Brigham and Women's Hospital, Boston, Massachusetts, USA; 2 Translational Medicine, GI Drug Discovery Unit, Takeda Pharmaceuticals, Cambridge, Massachusetts, USA

**Keywords:** systematic review, screening, paediatrics, child health, public health

## Abstract

**Introduction:**

Eighty percent of neonatal deaths occur among babies born preterm and/or small for gestational age (SGA). In sub-Saharan Africa and South Asia, approximately 40% of births occur outside of health facilities, and gestational age (GA) and birth weight are commonly unknown. Foot length (FL) has been proposed as a simple, surrogate measurement to identify and triage small babies born in the community. We conducted a systematic review and meta-analysis of the diagnostic accuracy of newborn FL to classify preterm and low birthweight infants.

**Methods:**

PubMed, EMBASE, Cochrane, Web of Science, POPLINE and WHO Global Health Library databases were searched. Studies of live-born infants that compared FL with GA and/or birth weight were included. Data on diagnostic accuracy were summarised, described, and pooled, as appropriate.

**Results:**

Six hundred and two studies were identified and 41 included. Techniques for measuring FL included use of a firm plastic ruler, callipers, footprint or a measuring board. Twelve studies assessed the diagnostic accuracy of FL to identify preterm births; however, data were not pooled given heterogeneity and low quality of GA. 19 studies used FL to identify low birthweight infants (<2500 g, <2000 g). Among studies in Asia (n=3), FL <7.7 cm had pooled sensitivity and specificity of 87.6% (95% CI 61.1% to 99.0%) and 70.9% (95% CI 23.5% to 95.1%), respectively, to identify <2500 g infants. FL <7.3 cm had 82.1% (95% CI 63.7% to 92.2%) sensitivity and 82.1% (95% CI 59.2% to 90.8%) specificity for identifying <2000 g infants (n=3). In the African studies (n=3), FL <7.9 cm had pooled sensitivity and specificity of 92.0% (95% CI 85.6% to 95.7%) and 71.9% (95% CI 44.5% to 89.1%), respectively, to identify <2500 g neonates.

**Conclusions:**

FL is a simple proxy measure that can identify babies of low birthweight with high sensitivity, though somewhat lower specificity. Additional research is needed to determine the validity of FL to identify preterm infants, and understand the programmatic impact of screening on healthcare seeking and outcomes.

**PROSPERO registration number:**

CRD42015020499

**Key questions**

**What is already known?**

- An estimated 80% of neonatal deaths occur in small infants—either born preterm (<37 weeks gestation) and/or small for gestational age.
- Gestational age and birthweight measurement are challenging and often not available among infants born outside of health facilities in low-income and middle-income settings.
- Neonatal foot length has been used as a simple, feasible surrogate method for identifying high-risk infants to link them with facilities and special newborn care in these settings.

**What are the new findings?**

- The measurement of gestational age among studies of foot length was heterogeneous, with generally low quality (ie. not early ultrasound).
- In pooled analysis of the Asian studies, foot length size of <7.7 cm had 88% sensitivity and 71% specificity for identifying infants<2500 g, and foot length <7.3 cm had 82% sensitivity and 82% specificity for identifying <2000 g infants.
- In pooled analysis of the African studies, foot length size of <7.9 cm had sensitivity and 92% and specificity of 72% to identify <2500 g neonates.

**What do the new findings imply?**

- Measurement of foot length is a simple and low-cost screening tool with high sensitivity yet lower specificity to identify infants of low birthweight in community settings where birth weighing scales are unavailable.
- Additional research is needed to ascertain the validity of foot length to identify preterm infants, and to study the implementation and impact of programmes to identify and manage low birthweight infants at the community level.

## Introduction

Each year, an estimated 20.5 million newborns are born low birthweight (LBW) (<2500 g) worldwide, with nearly three-quarters occurring in South Asia and sub-Saharan Africa.^1^ LBW may result from preterm birth and/or small for gestational age (SGA), commonly defined as birth weight below the 10% for gestational age and sex. Complications of preterm birth are now the leading cause of mortality among children under 5, resulting in 1 million deaths annually.^2^ Small size at birth, due to either preterm birth or SGA, accounts for more than 80% of neonatal deaths worldwide.^3^ Thus, increasing attention has focused on the identification, triage and management of small babies in low-income and middle-income countries (LMIC) to reduce neonatal morbidity and mortality.

In sub-Saharan Africa and South Asia, approximately 40% of births occur without skilled birth attendants, and, although facility births are increasing, 30%–45% of births still occur in the community setting—and as high as 65% among women from rural areas and the poorest wealth quintile.^4^ The identification of preterm and SGA babies in these settings is challenging. Many babies in LMIC are not weighed at birth,^1^ both in home births and in primary health facilities where weighing scales are not available and/or staff are overburdened. In 2015, 40 million (one-third) of babies born globally had no recorded birthweight, 97% of whom were in Asia and Africa.^5^ Furthermore, in the majority of settings in LMIC, GA of the pregnancy is often uncertain or unavailable.^6^ Last menstrual period may be unknown or affected by poor recall,^7 8^ and ultrasonography is not available, or is only available late in pregnancy, when traditional ultrasound has been less accurate for GA dating.^7–9^

The early and accurate identification of small and preterm infants in these settings is the first step to providing these high-risk babies with potentially life-saving interventions. This was recognised as a global priority in *The Global Action Report on Preterm Birth*—a collaboration between March of Dimes, the Partnership for Maternal, Newborn and Child Health, Save the Children and the WHO.^10^ Thus, it is critical to identify accurate screening techniques that are simple, low cost and feasible, which could be adopted in LMIC settings. Surrogate neonatal anthropometric measures, such as mid-upper arm, chest and head circumferences, have been tested for identifying LBW and/or preterm newborns.^11–13^ For this review, we chose to focus on neonatal foot length, which has emerged as a programmatically useful method to identify small babies in LMIC. Measurement of the foot can be done with locally available, portable, low-cost tools; does not require heavy or specialised equipment (eg, weighing scales or circumference tape measures); and the foot is easy to access without requiring undressing or unwrapping the baby.

The aim of this study was to systematically review the evidence for the diagnostic accuracy of foot length as a measure to identify high-risk preterm and LBW babies.

## Methods

### Search strategy

**Figure 1:**
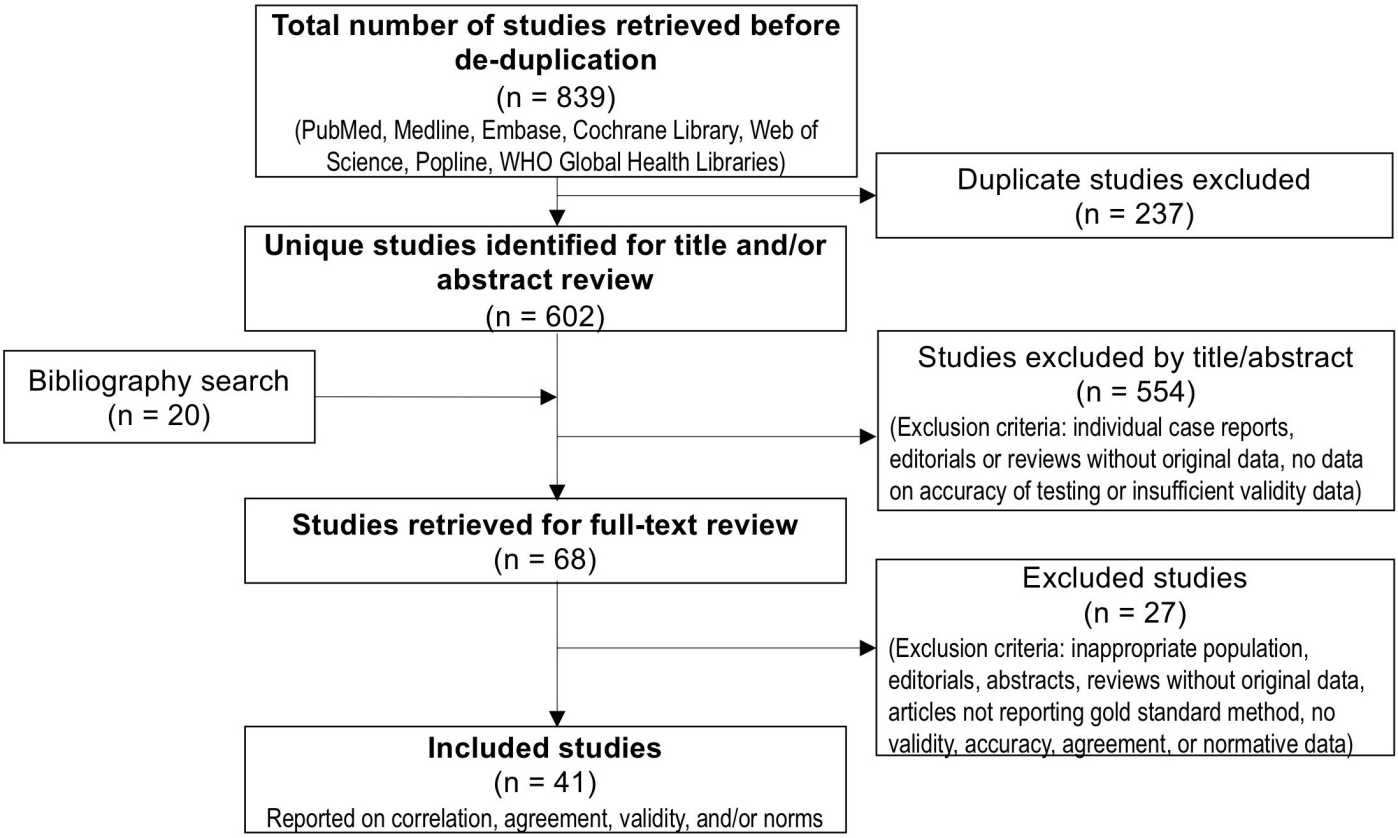
Foot length flow diagram. Diagram of the screening process to identify studies for inclusion in foot length review; adapted from Moher *et al*^14^).

We conducted a systematic review of the published literature. The searches were initially performed in May 2015 and updated in May 2020 (figure 1). The Preferred Reporting Items for Systematic Reviews and Meta-Analyses^14^ (PRISMA) statement and review protocol are available in the online supplemental appendix (online supplemental web appendix 1–2). The following databases were searched: PubMed, Embase, Cochrane, Web of Science, Popline and the WHO Global Health Libraries/regional databases (Latin American and Caribbean Health Sciences Literature (LILACS), Index Medicus for South East Asia Region (IMSEAR), Western Pacific Regional Office (WPRO), Index Medicus for the Eastern Mediterranean Region (IMEMR), Africa Index Medicus (AIM). Detailed search terms are available in online supplemental web appendix 3.

10.1136/bmjgh-2020-002976.supp1 Supplementary data

### Inclusion criteria

There were no language restrictions. Non-English abstracts were translated to English to determine if they met inclusion criteria, and relevant full-text articles were then translated to English using Google Translate or a fluent speaker.

Articles were considered for inclusion if the study: (1) included live-born neonates, (2) included data on newborn foot length and either GA or birthweight and (3) reported at least one statistic comparing foot length with GA or birth weight or reported on inter-rater or intra-rater reliability. Preterm births were defined as live-born infants born at <37 weeks gestation. We included data on validity for the identification of preterm infants, infants <2500 g (LBW), and <2000 g.

### Exclusion criteria

We excluded individual case reports, duplicate studies and studies of specialised subpopulations or selected populations. We also excluded studies that reported on fetal deaths or ultrasound-measured/fetal foot length.

### Data extraction

Data were extracted on study setting, design, population characteristics, participant selection, foot length measurement technique, GA or birth weight measurement, correlation, inter-rater and intra-rater reliability, and validity of foot length to identify preterm and/or LBW infants (<2500 g and/or <2000 g) (online supplemental web appendix 2). Two independent reviewers conducted data extraction, and differences were resolved by a third reviewer. For studies reporting diagnostic accuracy, data on the validity of all reported or published foot length cut-offs were extracted. One study reported vertical foot length distance,^15^ and this was converted to an equivalent heel-hallux distance using methods reported in online supplemental web appendix 4.

### Study quality assessment

For studies reporting diagnostic accuracy, methodological quality was assessed per the Cochrane Diagnostic Test Accuracy working group recommendations using the Quality Assessment of Diagnostic-Accuracy Studies (QUADAS-2),^16^ which was modified to fit the context of this study (online supplemental web appendix 2, section 5). Individual studies were evaluated for limitations and biases in the following five domains: study design, population selection and representativeness, definitions, precision and generalisability to the population of interest. The study-level QUADAS-2 score sheets are available on request.

### Statistical analysis

Stata V.15 (StataCorp) was used for data analysis. Studies were summarised and stratified for analysis by major WHO geographical region (ie, Asia, Africa). The regional grouping is based on the approach used by the Child Health Epidemiology Reference Group^17^ and hypothesised differences in birth size and foot length cut-offs between regions. Studies with comparable foot length thresholds were grouped. Studies reported results differently with respect to inclusion of the specific cut-off value (ie, <or < the threshold). For the practicality of health worker interpretation and future implementation, we grouped studies with cutoffs as less than or equal to (<) a particular foot length cut-off. For example, assuming a precision of 0.1 cm (1 mm), we grouped studies that analysed a <7.4 cm cut-off together with those that used a<7.5 cm cut-off.

Outcomes of interest for pooled diagnostic accuracy analysis included preterm birth, birthweight <2500 g and <2000 g. For pooled analysis with adequate study numbers, hierarchal models were used to pool sensitivity and specificity using the STATA metandi command. For those with <4 studies, proportions were logit transformed and standard errors calculated. Meta-analysis was conducted with random effects using the method of DerSimonian and Laird.^18^

### Patient and public involvement

This study did not involve any patients.

## Results

Six hundred and two unique studies were identified in the searches, and 68 full-text studies were reviewed. Forty-one studies were extracted and included in the final review (figure 1). Among these, 19 studies reported diagnostic accuracy data. Nine studies reported data on the diagnostic accuracy of foot length for identifying both preterm births and LBW infants (<2500 g or <2000 g), 3 papers reported only on preterm, and 7 papers reported only on LBW. The remaining papers (n=22) reported either data on the correlation between foot length and GA or birth weight, normal values of foot length for different GAs, and/or inter-rater or intra-rater reliability.

### Overall study characteristics

The basic study characteristics of all included studies are shown in online supplemental web appendix 5. Thirty-five were in LMIC (25 in Asia, 10 in sub-Saharan Africa), with the remainder in high-income countries (3 in Europe, 3 in North America). Twenty-nine studies were conducted in health facilities/hospitals, seven were community based and the rest were not specified.

### Study quality

The overall QUADAS-2 summary figure for all included studies with diagnostic accuracy data (n=19) is shown in online supplemental web appendix 6. In general, the quality of the studies was relatively low. There was a high risk of bias in over half of the studies related to patient selection and reference standard. Many studies were conducted in neonatal intensive care units (NICUs), which may affect the generalisability of the screening tool’s diagnostic accuracy in the general population of newborns. Risk of bias from the reference standard was also high in most studies assessing foot length and GA, given that only two studies had an early ultrasound-based reference, with the remaining using a clinical newborn examination or last menstrual period. The individual study QUADAS-2 data are available on request.

### Foot length measurement techniques

Among the studies identified, investigators reported several different techniques and tools for measuring foot length (table 1; online supplemental web appendix 7). The physical distances measured included: (1) the maximal heel-to-hallux distance (base of the heel to tip of the hallux), (2) distance from base of the heel to the tip of the longest digit, and (3) maximal perpendicular vertical distance. Measurement tools included a firm ruler, sliding callipers, flexible measuring tape, a foot length measuring board, and, lastly, foot print and post hoc measurement on paper.

**Table 1:** Foot length measurement methods

Measurement tools	
Firm ruler (plastic, metal, wooden) (See online supplemental WebAppendix 7a)	Firm; low cost and locally accessible; does not required specialised tool or procurement.
Sliding callipers	Precise however requires specialised tool, more costly and difficult to procurethan ruler.
Flexible measuring tape	Low cost, locally available. Flexible tape is not fixed or firm; may be less reliable and prone to variation between measurements.
Footprint (See online supplemental WebAppendix 4a: eFigure 1)	Requires firm surface. Can be measured retrospectively. Challenges include local/cultural beliefs regarding foot/finger prints and requires cleaning foot afterwards.
Foot length measuring board (See online supplemental WebAppendix 7b)	Precise, reliable; however, requires specialised tool that maybe difficult to manufacture or procure.
**Measurement Techniques/Axis**	
Heel-to-hallux	Linear distance measured from the base of the heel to the tip of hallux (big toe)
Heel-to-longest toe	Linear distance measured from the base of the heel to the tip of the longest toe (first, second, or third digit)
Vertical distance	Linear distance measured from the base of the heel to longest digit, along vertical axis of foot

### Normal distribution of foot length by GA

Nine studies were identified that reported the normal distribution of foot length by GA, which are shown in table 2 by region. Of those, six were from Asia,^19–24^ two from Europe,^25 26^ and one from North America.^27^

**Table 2:** Normative foot length data by gestational age (GA)

Author (year)	Country	GA reference standard	Foot length distance measured	Mean foot length for GA, cm (SD)		
				28 weeks	34 weeks	37 weeks
**Asia**						
Kulkarni^21^ (1992)	India	LMP and Dubowitz score	Heel-to-hallux	5.66 (0.68)	6.70 (0.64)	6.99 (0.56)
Kabra^20^ (1989)	India	LMP and Dubowitz score	Heel-to-hallux	5.28 (0.70)	6.83 (0.38)	7.55 (0.54)
Mathur^22^ (1984)	India	LMP	Heel-to-hallux	5.69 (0.35)	6.93 (0.44)	7.26 (0.34)
Singhal^23^ (2014)	India	LMP and Extended New Ballard Score	Heel-to-longest toe	5.45 (0.26)	6.80 (0.21)	7.53 (0.28)
Srivastava^24^ (2015)	India	Extended New Ballard Score	Heel-to-longest toe	5.50 (0)	6.54 (0.24)	7.45 (0.13)
Rakkappan^19^ (2016)	India	-	-	5.26	6.11	-
**Europe**						
Merlob^25^ (1984)	Israel	LMP, Dubowitz score, and anterior vascular capsule of the lens examination	Heel-to-hallux	5.25 (0.53)	6.81 (0.70)	7.53 (0.60)
Vocel^26^ (1978)	Czechoslovakia	LMP	Heel-to-hallux	-	6.83 (0.31)	7.23 (0.50)
**Americas**						
Usher^27^ (1969)	Canada	LMP	Heel-to-longest toe	5.54 (0.31)	6.96 (0.38)	7.80 (0.39)

(-) symbol indicates that data is not available for that paper.

LMP, last menstrual period.

### Correlation of foot length with GA and birth weight

Seventeen studies reported on the correlation of foot length with GA, with correlation coefficients ranging from 0.093 to 0.99 (median 0.873; n=14 studies) (online supplemental web appendix 8A). One of the two studies that had an ultrasound-based reference reported the lowest correlation coefficient (0.093), though included a narrow range of GA.^28^ The majority of studies (n=13) with GA data used neonatal clinical assessment as the reference standard, eight of which reported correlation coefficients greater than 0.85.

Twenty-one studies reported on the correlation between birthweight and foot length (13 Asia, 6 Africa, 1 Europe) (online supplemental web appendix 8B). Correlation coefficients ranged from 0.213 to 0.951 (median 0.755; n=14 studies). Data were not pooled on correlation coefficients due to the lack of reporting on the type of correlation coefficient (ie, Spearman vs Pearson) for a majority of studies.

### Diagnostic accuracy of foot length to identify preterm births

We identified 12 studies that assessed the diagnostic accuracy of foot length to classify preterm infants (Asia n=8, Africa n=4) (table 3). Eight studies reported areas under the curve (AUCs) for identifying infants <37 weeks, which ranged from 0.52 to 0.89 in 5 South Asian studies,^12 28–31^ and from 0.86 to 0.95 in 3 African studies^32–34^ (table 3). The eight Asian studies used different methods of reference standard GA determination. Five studies used a postnatal clinical exam (New Ballard Score, NBS) as the reference standard, and reported relatively high diagnostic accuracy.^12 23 31 35 36^ On the other hand, the three Asian studies that used an LMP or ultrasound-based reference standard GA reported lower sensitivity and specificity.^28–30^ Similarly, three studies from Africa used a postnatal clinical exam (NBS and/or Eregie) as the GA reference standard, and reported relatively higher diagnostic accuracy.^32 34 37^ One 2019 study conducted in rural Tanzania, which used an ultrasound-based reference standard GA, reported comparably high diagnostic accuracy^33^—higher than that of the one other study (in Bangladesh) that had ultrasound dating.^28^

**Table 3:** Diagnostic accuracy of foot length to identify preterm (<37 week) infants, by world region

Author (year)	Country	GA reference standard	N	% Preterm	Definition of preterm (weeks)	Foot length cut-off (cm)	Sensitivity (%)*	Specificity (%)*	PPV (%)	NPV (%)	AUC*
**Asia**											
Lee^28^ (2016)†	Bangladesh	Ultrasound	710	8.3	<37	<7.5	64	35	8	92	0.5191
KC^29^ (2015)†	Nepal	LMP	811	6.7	<37	7.8	76.9	53.9	10.6	97.0	0.683 (95% CI 0.610 to 0.756)
Pratinidhi^30^ (2017)	India	LMP	645	6.7‡	<34	<6.8	93.0 (95% CI 80.9 to 98.5)	86.7 (95% CI 83.7 to 89.3)	–	–	0.943
				16.1‡	<37	<7.0	81.7 (95% CI 73.0 to 88.6)	80.8 (95% CI 77.2 to 84.0)	–	–	0.891
Singhal^23^ (2014)	India	LMP and ‘extended’ NBS	1000	15.4‡	<34	7	94.76	94.3	81.55	98.54	–
Mukherjee^35^ (2013)†	India	NBS	351	48.1	<37	<7.75	92.3	86.3	–	–	–
Roy^31^ (2019)	India	NBS	320	17.5	<37	7.35	80	78	–	–	0.772
Srinivasa^36^	India	NBS	500	16.8	<37	<7.4	98.81 (95% CI 93.5 to 100.0)	79.09 (95% CI 74.9 to 82.9)	–	–	–
Thi^12^ (2015)	Vietnam	NBS	485	47	<37	<7.3	80 (95% CI 74 to 85)	81 (95% CI 76 to 86)	79 (95% CI 73 to 84)	82 (95% CI 77 to 87)	0.88 (95% CI 0.85 to 0.91)
**Africa**											
Gidi^34^ (2020)	Ethiopia	NBS	1389	10.2	<37	<7.5	81.7 (95% CI 74.3 to 87.7)	77.0 (95% CI 74.6 to 79.3)	28.6 (95% CI 24.3 to 33.3)	97.4 (95% CI 96.2 to 98.3)	0.86 (95% CI 0.84 to 0.88)
		Eregie examination	1389	5.5	<37	<7.4	80.5 (69.9–88.7)	91.4 (89.7–92.8)	35.2 (28.2–42.8)	98.8 (98.0–99.3)	0.93 (0.91–0.94)
Marchant^37^ (2010)	Tanzania	Eregie examination	529	9	<37	<8	93 (95% CI 82 to 99)	58 (95% CI 53 to 62)	15	99	–
Nabiwemba 32 (2013a)†	Uganda	Eregie examination	711	4	<37	7.5	85.7	90.4	27.0	99.4	0.95
						8.0	96.4	66.2	10.6	99.8	
Paulsen 33 (2019)	Tanzania	Ultrasound	376	4.5	<37	<7.45	88 (95% CI 64 to 99)	85 (95% CI 81 to 88)	21 (95% CI 13 to 33%)	99 (95% CI 98 to 100%)	0.9358
						<7.7	94 (95% CI 71 to 100)	64 (95% CI 59 to 69)	11 (95% CI 6 to 17%)	100 (95% CI 98 to 100%)	

(-) symbol indicates that data is not available for that paper.

*Sensitivity/specificity data were converted to a % if in decimal form, and AUC was converted to decimal form if reported as a per cent.

†Paper also reported diagnostic accuracy data for other foot length cut-offs.

‡Per cent calculated by authors (LVF, PP and ACL) from data reported in the paper.

AUC, area under the curve; GA, gestational age; LMP, last menstrual period; NBS, New Ballard Score; NPV, negative predictive value; PPV, positive predictive value; US, ultrasound.

### Diagnostic accuracy of foot length to identify LBW infants (<2500 g)

We identified 15 cohorts in which the diagnostic accuracy of foot length to identify <2500 g infants was assessed (Asia n=8, Africa n=7) (online supplemental web appendices 9, 10A, B). Seven reported AUCs, which ranged from 0.84 to 0.94 in the two Asian studies,^12 15^ and 0.74 to 0.97 in the five African studies^32–34 38 39^ (online supplemental web appendix 9).

Diagnostic accuracy data were pooled for the identification of infants<2500 g for several cutoffs (table 4). Among the Asian studies, a foot length cut-off of <7.7 cm had a pooled sensitivity of 87.6% (95% CI 55.7% to 97.5%) and specificity of 70.9% (95% CI 23.5% to 95.1%) (n=3 studies) (table 4).^15 35 40^ Among the African studies, the pooled sensitivity and specificity for a foot length cut-off of <7.9 cm were 92.0% (95% CI 85.6% to 95.7%) and 71.9% (95% CI 44.5% to 89.1%), respectively (n=3 studies) (table 4).^32 37 41^

**Table 4:** Pooled sensitivity and specificity for all available foot length thresholds to identify low birthweight neonates (<2500 g and <2000 g)

Birth weight cut-off	Foot length cut-off (cm)	N, for pooling	Pooled sensitivity (%) (95% CI)	Pooled specificity (%) (95% CI)
**Africa**				
<2500 g	<7.6	2	86.9 (82.9 to 90.2)	74.1 (58.6 to 85.3)
	<7.7	3	84.6 (80.3 to 88.2)	73.5 (46.7 to 89.8)
	<7.9	3	92.0 (85.6 to 95.7)	71.9 (44.5 to 89.1)
**Asia**				
<2500 g	<7.2	2	40.2 (27.9 to 53.9)	89.3 (67.3 to 90.8)
	<7.3	3	59.7 (37.9 to 78.3)	80.8 (57.2 to 93.0)
	<7.4	3	69.6 (43.9 to 87.0)	79.9 (55.7 to 92.6)
	<7.5	2	70.3 (42.3 to 88.5)	66.4 (32.7 to 88.9)
	<7.6	2	80.7 (55.2 to 93.5)	55.0 (23.2 to 83.3)
	<7.7	3	87.6 (55.7 to 97.5)	70.9 (23.5 to 95.1)
	<7.8	2	92.7 (61.1 to 99.0)	33.9 (11.2 to 67.7)
<2000 g	<6.8	4	58.4 (29.4 to 82.6)	96.0 (90.0 to 98.5)
	<6.9	3	48.9 (30.9 to 67.2)	95.4 (86.9 to 98.5)
	<7.0	3	57.7 (32.2 to 79.7)	93.0 (80.9 to 97.6)
	<7.1	3	67.5 (55.7 to 77.5)	87.8 (63.9 to 96.7)
	<7.2	3	79.6 (67.2 to 88.1)	85.7 (65.7 to 94.9)
	<7.3	3	82.1 (63.7 to 92.2)	82.1 (59.2 to 90.8)
	<7.4	3	85.1 (66.2 to 94.4)	76.0 (50.0 to 90.8)
	<7.5	3	88.6 (73.9 to 95.5)	69.4 (41.8 to 87.7)

### Diagnostic accuracy of foot length to identify infants <2000 g

Four studies reported on the diagnostic accuracy of foot length to identify infants <2000 g in Asia (online supplemental web appendix 10C).^15 29 30 40^ Two studies reported AUCs (0.88 and 0.93, respectively) (online supplemental web appendix 9).^15 29^ In the meta-analysis, a foot length cut-off of <7.3 cm classified <2000 g infants (table 4) with sensitivity of 82.1% (95% CI 63.7% to 92.2%) and specificity of 82.1% (95% CI 59.2% to 90.8%) (n=3 studies).^15 29 40^

### Inter- and intra-rater reliability

Eight studies were identified that compared the agreement of repeated foot length measurements between and/or within assessors (online supplemental web appendix 11). Five studies were conducted in Asia,^15 35 42–44^ two in Africa,^45 46^ and one in Europe.^47^ Four studies reported on interobserver kappa to classify small feet, which ranged widely from 0.30 to 0.82.^35 44–46^ Of the eight studies, the majority (n=5) were in hospital settings. Measurements were conducted by medical staff (physicians, nurses, midwives) in three studies,^35 45 47^ while another three studies included measurements by community volunteers, caretakers or field workers^15 44 46^ (not reported in two studies). Three studies were in community settings, and two compared foot length measured by a healthcare worker to that of a community volunteer or caretaker, reporting kappa statistics of 0.53 and 0.82.^44 46^ One study in Tanzania found that community volunteers systematically undermeasured foot length by a mean of 0.26 cm compared with researchers.^46^ Four studies reported on intra-rater reliability, with coefficients of variation ranging from 1.05%^47^ to 1.56%.^42^ Another study reported a within-infant range of measures of <0.2 cm in 98.4% of infants.^15^

## Discussion

Improving the identification and care of small, high-risk babies is essential to reduce the global burden of neonatal morbidity and mortality. Given that GA and birth weight information is commonly missing in half of births in sub-Saharan Africa and South Asia,^1 6^ foot length measurement has emerged as a promising method to identify vulnerable infants born in community settings. In this systematic review and meta-analysis, we found that foot length thresholds of <7.7 cm in Asia and <7.9 cm in Africa classified LBW (<2500 g) infants with high sensitivity and lower specificity, and foot length <7.3 cm had relatively high sensitivity and specificity (>80%) to classify infants <2000 g. Data assessing the accuracy of foot length for identifying preterm infants were limited by both quality and heterogeneity of reference standard GA dating method.

Different methods of foot length measurement have been described in the literature. Some investigators have used specialised or higher cost equipment, such as customised measuring boards^15 22 42 47^ or callipers.^11 19 21 48^ The most common method used across studies was the measurement of the heel-to-hallux (or to longest digit) with a firm ruler. This method is low cost, easy to train and feasible at the community level. Two studies compared the diagnostic accuracy of different measuring techniques (firm plastic ruler, measuring tape, footprint), and both found that the firm ruler had the highest predictive score for identifying both preterm and LBW newborns.^29 32^ Feasibility, training, standardisation and cost of equipment are key considerations for scalability in LMIC. In particular, standardising the landmarks used in foot length measurement is critical. The majority of studies that reported normative values for foot growth used the maximum heel-hallux distance,^20–22 25 26^ though several used the heel-to-longest toe distance.^23 24 27^ Having standardised landmarks for the distance measured is essential for both consistency of foot length measurements and comparisons between populations.

In the studies included in this review, data on the accuracy of foot length to identify preterm births were heterogeneous and generally of low quality. Only two studies used an early ultrasound-based reference standard GA,^28 33^ while most relied on clinical exam to determine GA, which estimates GA within ±4 weeks of ultrasound dating.^49^ The Eregie examination was commonly used in Africa. In this simplified examination, newborn anthropometrics (head circumference and mid-upper arm circumference) are included, and thus, dating is strongly influenced by newborn size.^50^ In a systematic review, out of three studies assessing the diagnostic accuracy of the Eregie examination, only one used an ultrasound reference and found that the Eregie dated pregnancies within ±3.5 weeks of ultrasound dating.^28 49^ In this review, among the Asian studies, the range of diagnostic accuracy ranged widely (sensitivity: 64%–98%, specificity: 35%–94%), which may be due to the variation of reference standard GA dating methods, or potentially due to the challenge of discriminating SGA versus preterm infants in settings with high prevalence of fetal growth restriction. In South Asia, this prevalence is as high as 30%. In addition, neonatal clinical examinations (used to determine reference GA in five of the eight Asian studies) have been shown to systematically underestimate GA among growth-restricted infants.^49^

Foot length was a reasonable proxy of infant size to identify LBW infants. The foot length thresholds to classify LBW were lower in Asia, where babies are smaller and SGA is more prevalent.^1 51^ For studies in Asia, a foot length cut-off of <7.7 cm identified <2500 g infants with pooled sensitivity of 87.6% and specificity of 70.9%; for identifying <2000 g infants in Asia, a foot length cut-off of <7.3 cm had 82.1% sensitivity and 82.1% specificity. In Africa, a foot length cut-off of <7.9 cm had a pooled sensitivity of 92.0% and specificity of 71.9% to identify <2500 g infants. The balance of sensitivity and specificity is a critical consideration in health systems that must weigh the increasing demand generated by identifying and referring more high-risk babies with the supply of available services and the risk of overburdening health systems. Based on our data, if a community-based foot length screening programme was implemented to refer LBW (<2500 g) infants in South Asia, where the prevalence of LBW is 30%,^1^ in a population of 100 000 newborns, there would be 26 400 LBW infants correctly identified, 3600 LBW babies missed and 20 300 non-LBW babies who were over-referred (false positives). Approximately 57% referred to health facilities would be truly LBW, and 93% of babies with foot length >7.8 cm would not be LBW. In sub-Saharan Africa, where the prevalence of LBW (<2500 g) is 16.4%,^1^ in a population of 100 000 newborns, 15 088 LBW babies would be correctly identified, 1312 LBW babies would be missed and 23 408 over-referred. Approximately 40% of referred babies would be LBW, and 98% of babies with foot length >8.0 cm would not be LBW. The local health system and public health implications should be considered for the implementation of any such screening programmes.

Training and standardisation are important considerations for programmatic implementation in LMIC. Intra-rater and inter-rater agreement was generally high for neonatal foot length measurement. Foot length measurement is advantageous, as it can be easily performed with minimal medical training. Two studies assessed inter-rater agreement between a healthcare provider or researcher and a lay community health worker or caretaker, a comparison of important programmatic relevance.^44 46^ In Tanzania, Marchant *et al* reported that community volunteers systematically undermeasured foot length compared with research staff and overestimated those needing special care in the community.^46^ Reliability and continued quality assurance of measurements are important considerations for the potential scale up of this tool in programmatic and research settings, especially considering reliability in a variety of users.

There are several important limitations to this review. The overall quality of studies included in the review was low, with limitations in the quality of reference standard GA data, reporting and selection bias. There is a need for more studies with high-quality ultrasound dating or best obstetric estimate as the reference standard. In addition, we did not put a date restriction on our studies, as many of the original foot length articles were published in the 1970s. However, all studies that reported diagnostic accuracy data and were included in the meta-analyses were from after 2000, with the majority published after 2010. We conducted pooled analysis by major WHO world region, though countries within these regions are heterogeneous and optimal foot length cutoffs may vary by country. Finally, we limited the scope of this review to diagnostic accuracy only, and it would be valuable to assess the effect of foot length measurement as a screening tool on referrals, care seeking behaviours and infant health outcomes. We are aware of an upcoming study in Nepal^52^ that will assess these outcomes.

## Conclusions

In summary, improving the identification of small babies at the community level is a critical first step to triage these high-risk infants and provide timely and potentially life-saving interventions. Foot length is a low-cost, simple and feasible measurement, with potential to identify LBW infants in low-resource communities. Standardisation of landmarks and measurement techniques is important. More studies are needed with accurate GA dating to determine the diagnostic accuracy of foot length measurement as a screening tool to identify preterm infants in LMIC. Given the lower specificity and potential for over-referral, alternative surrogate measures with higher specificity should also be sought and studied. Finally, programmatic and implementation research is needed to determine the effect of such screening programmes on newborn care seeking and health outcomes.
