# Supplementary material for: Diagnostic accuracy of neonatal foot length to identify preterm and low birthweight infants: a systematic review and meta-analysis
Source: BMJ Glob Health. 2020 Nov 18;5(11):e002976. doi: 10.1136/bmjgh-2020-002976 (PMC7677351; doi:10.1136/bmjgh-2020-002976)
Supplement: Supplementary data [file bmjgh-2020-002976supp001.pdf]

WEBAPPENDIX

Diagnostic accuracy of neonatal foot length to identify preterm and low birth weight infants: a systematic review and meta-analysis

Table of Contents

WebAppendix 1: PRISMA Statement 2

WebAppendix 2: Systematic Review Protocol……………………….……...….….…. 4

WebAppendix 3: Search Terms…………………………………….…….……...……...8

WebAppendix 4a-b: Conversion of Vertical to Heel-Hallux Foot Length Distance.…9

WebAppendix 5: Overall Study Table…………………………….……………..……...…..11

WebAppendix 6: QUADAS-2 Summary…………………………...……………………13

WebAppendix 7: Measurement Method Figures……………......................................14

WebAppendix 8a-b: Correlation between Foot Length and GA/Birthweight.............15

WebAppendix 9: Areas Under the Curve for Foot Length…………………………..…18

WebAppendix 10a-c: Diagnostic Accuracy of Foot Length for Birthweight…….…….19

WebAppendix 11: Inter-rater Reliability Data…………………………..……………....22

WebAppendix References…………………………………………………………...…….23

Folger LV, *et al. BMJ Global Health* 2020; 5:e002976. doi: 10.1136/bmjgh-2020-002976

WebAppendix 1. PRISMA Statement.

Preferred Reporting Items for Systematic Reviews and Meta-Analyses (PRISMA), 2009[1]

Section/topic	#	Checklist item	Reported on page #
**TITLE**			
Title	1	Identify the report as a systematic review, meta-analysis, or both.	1
**ABSTRACT**			
Structured summary	2	Provide a structured summary including, as applicable: background; objectives; data sources; study eligibility criteria, participants, and interventions; study appraisal and synthesis methods; results; limitations; conclusions and implications of key findings; systematic review registration number.	3
**INTRODUCTION**			
Rationale	3	Describe the rationale for the review in the context of what is already known.	5-6
Objectives	4	Provide an explicit statement of questions being addressed with reference to participants, interventions, comparisons, outcomes, and study design (PICOS).	6
**METHODS**			
Protocol and registration	5	Indicate if a review protocol exists, if and where it can be accessed (e.g., Web address), and, if available, provide registration information including registration number.	7; Web Appendix 2
Eligibility criteria	6	Specify study characteristics (e.g., PICOS, length of follow-up) and report characteristics (e.g., years considered, language, publication status) used as criteria for eligibility, giving rationale.	7-8; Web Appendix 2.3
Information sources	7	Describe all information sources (e.g., databases with dates of coverage, contact with study authors to identify additional studies) in the search and date last searched.	7; Web Appendix 2.3
Search	8	Present full electronic search strategy for at least one database, including any limits used, such that it could be repeated.	Web Appendix 3
Study selection	9	State the process for selecting studies (i.e., screening, eligibility, included in systematic review, and, if applicable, included in the meta-analysis).	7-9; Figure 1; Web Appendix 2.3
Data collection process	10	Describe method of data extraction from reports (e.g., piloted forms, independently, in duplicate) and any processes for obtaining and confirming data from investigators.	Web Appendices 2.4 & 2.5
Data items	11	List and define all variables for which data were sought (e.g., PICOS, funding sources) and any assumptions and simplifications made.	Web Appendix 2.4
Risk of bias in individual studies	12	Describe methods used for assessing risk of bias of individual studies (including specification of whether this was done at the study or outcome level), and how this information is to be used in any data synthesis.	8-9; Web Appendix 2.5
Summary measures	13	State the principal summary measures (e.g., risk ratio, difference in means).	9; Web Appendix 2.6

Section/topic	#	Checklist item	Reported on page #
Synthesis of results	14	Describe the methods of handling data and combining results of studies, if done, including measures of consistency (e.g., I^2) for each meta-analysis.	9; Web Appendix 2.6
Risk of bias across studies	15	Specify any assessment of risk of bias that may affect the cumulative evidence (e.g., publication bias, selective reporting within studies).	Web Appendix 2.7
Additional analyses	16	Describe methods of additional analyses (e.g., sensitivity or subgroup analyses, meta-regression), if done, indicating which were pre-specified.	N/A
**RESULTS**			
Study selection	17	Give numbers of studies screened, assessed for eligibility, and included in the review, with reasons for exclusions at each stage, ideally with a flow diagram.	10; Figure 1
Study characteristics	18	For each study, present characteristics for which data were extracted (e.g., study size, PICOS, follow-up period) and provide the citations.	10; Web Appendix 5
Risk of bias within studies	19	Present data on risk of bias of each study and, if available, any outcome level assessment (see item 12).	10-11; Web Appendix 6
Results of individual studies	20	For all outcomes considered (benefits or harms), present, for each study: (a) simple summary data for each intervention group (b) effect estimates and confidence intervals, ideally with a forest plot.	11-14, 26-27; Web Appendices 8a-b, 9, 10a-c, 11
Synthesis of results	21	Present results of each meta-analysis done, including confidence intervals and measures of consistency.	13, 28
Risk of bias across studies	22	Present results of any assessment of risk of bias across studies (see Item 15).	N/A
Additional analysis	23	Give results of additional analyses, if done (e.g., sensitivity or subgroup analyses, meta-regression [see Item 16]).	N/A
**DISCUSSION**			
Summary of evidence	24	Summarize the main findings including the strength of evidence for each main outcome; consider their relevance to key groups (e.g., healthcare providers, users, and policy makers).	15-18
Limitations	25	Discuss limitations at study and outcome level (e.g., risk of bias), and at review-level (e.g., incomplete retrieval of identified research, reporting bias).	18
Conclusions	26	Provide a general interpretation of the results in the context of other evidence, and implications for future research.	18-19
**FUNDING**			
Funding	27	Describe sources of funding for the systematic review and other support (e.g., supply of data); role of funders for the systematic review.	1

WebAppendix 2. Systematic Review Protocol

Diagnostic Accuracy of Methods of Gestational Age Determination
Systematic Review Protocol

1. Background

Preterm birth is the leading cause of under-5 child mortality. However, ascertainment of gestational age is limited and challenging in low resource settings. The accurate determination of gestational age in pregnancy and after birth is required in order to identify prematurity and fetal growth restriction, and effectively deliver interventions. The aim of this review is to identify a range of methods currently used to determine gestational age before and after birth, assess the validity of these methods, and identify potential new methods for application in low- and middle-income countries (LMIC).

2. Research questions

1) What range of methods are currently available to determine gestational age both before and after birth?
2) What are the accuracy, reliability, precision (i.e. validity) of these methods to assess gestational age?
3) What methods are available which are currently feasible for LMIC settings?
4) What new methods may be applicable to LMIC in the future?

3. Search Strategy

We will conduct automated and manual searches including multiple search engines and databases (Table 1). The databases will include: PubMed, Embase, Web of Science, Popline, Cochrane Library, Global Health Library, WHO regional database, www. clinicaltrials.gov and targeted Google searches. There will be no restrictions on language or publication period. The detailed search terms are listed in the Appendix formatted for PubMed.

Table 1: Databases and Search engines

Database	Website
PubMed/Medline	http://www.ncbi.nlm.nih.gov/pubmed
Embase	http://www.embase.com/
Web of Science	
Popline	
The Cochrane Library	http://www.cochrane.org/
Global Health Library	http://www.globalhealth.org/
World Health Organization Regional Databases: LILACs, IMEMR, AIM, IMSEAR, WPRIM	www.who.int
Clinical trials	www.clinicaltrials.gov
Targeted Google searches	

Selection Criteria

a. Inclusion Criteria:
   i. For Broader Landscape Review:
      1. All Inclusive
      2. Any Birth: Live births OR stillbirths
   ii. For Inclusion for diagnostic accuracy
      1. Comparison of at least 2 GA estimation methods
      2. Report on at least one statistic assessing accuracy of GA determination method
b. Exclusion Criteria:
   i. No language exclusions
   ii. Individual case reports
   iii. Duplicate studies
   iv. Editorials or reviews without original data
   v. No data on accuracy of testing or insufficient data to calculate

Figure 1: Sample Flowchart for Literature Searches

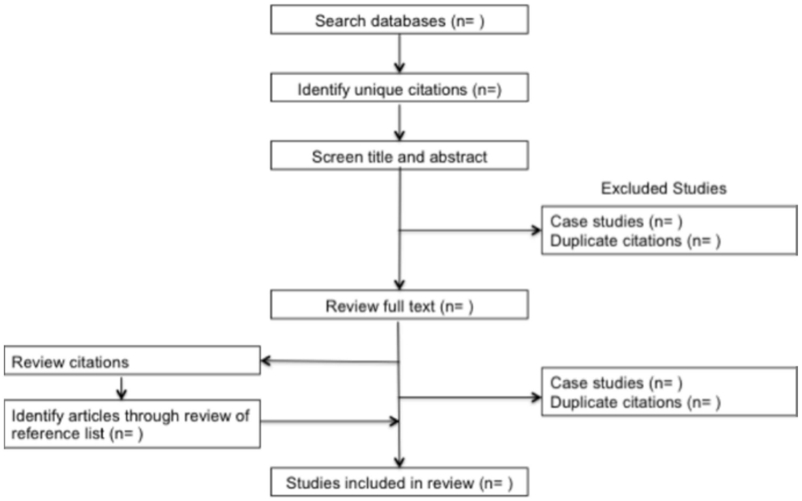

BMJ Publishing Group Limited (BMJ) disclaims all liability and responsibility arising from any reliance placed on this supplemental material which has been supplied by the author(s)

4. Data Abstraction

Data will be extracted into a standard Excel file by two independent reviewers. A sample of the variables to be extracted is shown in Table 2 (full list available in Web Appendix 4). As data are available, a two-by-two table will be constructed for each study to determine the true positives, false positives, true negatives, and false negatives, comparing the test method to the reference standard definition.

Table 2. Variables in Data Abstraction Table

Study Characteristics	Reference Standard GA Determination Method	Test GA Estimation Method(s)
• Authors<br>• Journal<br>• Publication year<br>• Country<br>• Study design<br>• Study setting<br>• Population characteristics<br>• Sample selection method<br>• Total sample size	• Type/description of method/ test<br>• Type/level of training of health worker performing assessment<br>• Mean GA [SD] of cohort with reference standard method<br>• Total number of preterm <37 weeks; preterm <34 weeks; LBW; SGA<br>• % preterm <37 weeks; preterm <34 weeks; LBW; SGA	• Type/description of reference standard and test methods<br>• Type/level of health worker performing assessments<br>• Mean GA (+ standard deviation) of cohort with reference standard and test methods<br>• Mean difference (+ standard deviation) between reference standard vs test method<br>• Total number or % of preterm <37 weeks; preterm <34 weeks; LBW; SGA<br>• Correlation coefficient with reference standard gestational age<br>• Area under the receiver operating curve<br>• Cutoff values (if applicable) with corresponding<br>o Sensitivity [95%CI] for preterm <37 weeks; preterm <34 weeks; LBW, SGA<br>o Specificity [95%CI] for preterm <37 weeks; preterm <34 weeks; LBW, SGA<br>o PPV for preterm <37 weeks; preterm <34 weeks; LBW, SGA<br>o NPV for preterm <37 weeks; preterm <34 weeks, LBW, SGA

CI= confidence interval, GA= gestational age, LBW= low birth weight, NPV= negative predictive value, PPV= positive predictive value, SD= standard deviation, SGA= small for gestational age

5. Study Quality Assessment

For studies reporting diagnostic accuracy, methodological quality will be assessed per the Cochrane Diagnostic Test Accuracy Working group recommendations using the QUADAS-2 (Quality Assessment of Diagnostic-Accuracy Studies-2).

All studies will be scored for quality by two independent researchers. If the data reviewers disagree, they will discuss their position in detail, using evidence from the study in question until they reach a compromise. If they do not reach a compromise, the question at hand will be discussed with the research team during a team meeting to arrive at a compromise that the team as a whole agrees with.

Methodological quality will be assessed per the Cochrane Diagnostic Test Accuracy Working Group's recommendations using the QUADAS-2 (Quality Assessment of Diagnostic Accuracy Studies-2). Individual studies will be evaluated for limitations and biases in the following domains: patient selection, reference standard method, test method, and flow and timing of the study. For each of these domains, a score will be assigned (0=low risk, 1=high risk). A total quality assessment score will be given to each study. Study design will be scored according to whether the sample size was sufficient (n≥50 vs. n<50), whether methodology and data were

Folger LV, *et al. BMJ Global Health* 2020; 5:e002976. doi: 10.1136/bmjgh-2020-002976

adequately reported, whether subjects were enrolled randomly vs. purposively, whether inappropriate exclusion criteria were avoided, whether the reference standard vs. test method were used independently and users were blinded, whether multiple measurements were taken to assess inter- and or/ intra-rater reliability, whether any quality control measures were undertaken, whether users were trained in the GA assessment method(s), whether thresholds were pre-specified (if applicable), whether the reference standard method was ultrasound (adequate) vs. other (inadequate) method, whether any enrolled subjects were excluded from assessment by either the reference standard method or the test method, whether all enrolled subjects received the same reference standard, and whether any enrolled subjects were excluded from the analysis.

In addition to summarizing study quality, we will also summarize the consistency of definitions of each gestational age method, and the overall generalizability of study results to our target population (newborns in LMICs).

6. Data Analysis

All data will be summarized in study data tables by each major group of methods of gestational age determination. If there is sufficient and adequate quality data to perform pooled analysis, we will conduct meta-analysis with hierarchal bivariate models using the Stata "metandi" command, as per the recommendations of the Cochrane Working Group on Systematic Reviews of Diagnostic Test Accuracy.[2] Hierarchal summary receiver operating characteristic curves will be generated with the "metanplot" command. Coupled forest plots will be generated with Review Manager 5.1. Sub-group analysis and meta-regression may be performed, if required, to explore sources of heterogeneity

7. Study Limitations

The potential limitations we foresee are the paucity of published studies. We therefore will attempt to target numerous search engines and sources in the grey and unpublished literature, as well as targeted Google searches. The study may potentially be limited if the studies found in our search are not representative of global regions.

8. Reporting

We plan to report these findings to public health experts in child and maternal health first by submitting interim and final reports to The Bill & Melinda Gates Foundation, and finally through publication in a peer-reviewed journal. Depending on the findings of the review, this may result in a publication supplement of 2-3 papers.

8. Protocol Registration

The protocol was registered in the PROSPERO International prospective register of systematic reviews, University of York Centre for Reviews and Dissemination (http://www.crd.york.ac.uk/PROSPERO). PROSPERO Registration number: CRD42015020499

Folger LV, *et al. BMJ Global Health* 2020; 5:e002976. doi: 10.1136/bmjgh-2020-002976

WebAppendix 3. Foot Length Search Terms

Date Searched	Database (report by each database searched)	Detailed Search Strings/MeSH Terms	# of hits per database
April 29, 2020	PubMed	"Gestational Age"[Mesh] OR "gestational age"[All Fields] OR "premature birth"[MeSH Terms] OR "premature birth"[All Fields] OR preterm[All Fields] OR "premature"[All Fields] OR prematurity[All Fields] OR ptb[All Fields] OR "fetal growth retardation"[MeSH Terms] OR "fetal growth restriction"[All Fields] OR "foetal growth restriction"[All Fields] OR "fetal growth retardation"[All Fields] OR "foetal growth retardation"[All Fields] OR "infant, low birth weight"[MeSH Terms] OR "low birth weight"[All Fields] OR "IUGR"[All Fields] OR "intrauterine growth restriction"[All Fields] OR "intrauterine growth retardation"[All Fields] OR "lbw"[All Fields] OR "birth weight"[MeSH Terms] OR "birth weight"[All Fields] OR "birthweight"[All Fields] OR "stillborn"[All Fields] OR "stillbirth"[All Fields] OR "fetal death"[All Fields] OR "foetal death"[All Fields] OR "fetal demise"[All Fields] OR "foetal demise"[All Fields] OR "menstrual age"[All Fields] OR "fetal age"[All Fields] OR "foetal age"[All Fields] OR "fetal growth"[All Fields] OR "foetal growth"[All Fields] OR "embryo growth"[All Fields] OR "fetal development"[All Fields] OR "foetal development"[All Fields] OR infant[MeSH] OR neonatal[Mesh]) AND ("foot length"[All Fields] OR "foot size"[All Fields] OR "foot measurement"[All Fields] OR "footlength"[All Fields] OR ("foot"[All Fields] AND anthropometr*[All Fields]) OR "foot measure"[All Fields]	208
April 29, 2020	Embase	(('gestational age'/exp OR 'gestational age' OR 'premature birth'/exp OR 'premature birth' OR 'preterm' OR 'premature'/exp OR 'premature' OR 'prematurity'/exp OR 'prematurity' OR 'ptb' OR 'fetal growth retardation') AND 'foetal growth retardation' OR 'foetal growth retardation' OR 'fetal growth restriction' OR 'foetal growth restriction' OR 'low birth weight' OR 'iugr' OR 'intrauterine growth retardation'/exp OR 'intrauterine growth retardation' OR 'intrauterine growth restriction' OR 'lbw' OR 'birth weight'/exp OR 'birth weight' OR 'birthweight'/exp OR 'birthweight' OR 'stillborn' OR 'stillbirth'/exp OR 'stillbirth' OR 'fetal death'/exp OR 'fetal death' OR 'foetal death'/exp OR 'foetal death' OR 'fetal demise'/exp OR 'fetal demise' OR 'foetal demise' OR 'menstrual age' OR 'fetal age' OR 'foetal age' OR 'fetal growth'/exp OR 'fetal growth' OR 'foetal growth'/exp OR 'foetal growth' OR 'embryo growth'/exp OR 'embryo growth' OR 'fetal development'/exp OR 'fetal development' OR 'foetal development'/exp OR 'foetal development' OR 'infant'/exp OR 'infant' OR 'neonatal') AND ('foot length' OR 'foot size' OR 'foot measurement' OR 'footlength' OR ('foot' AND anthropometr*) OR 'foot measure')	207
April 29, 2020	Cochrane	"foot length" or "foot size" or "foot measurement" or "footlength" or ("foot" and anthropometr*) or "foot measure" AND infant or baby	122
April 29, 2020	Web of Science	TS=("gestational age" OR "premature birth" OR preterm OR "premature" OR prematurity OR ptb OR "fetal growth retardation" OR "fetal growth restriction" OR "foetal growth restriction" OR "foetal growth retardation" OR "low birth weight" OR "IUGR" OR "intrauterine growth restriction" OR "intrauterine growth retardation" OR "lbw" OR "birth weight" OR "birthweight" OR "stillborn" OR "stillbirth" OR "fetal death" OR "foetal death" OR "fetal demise" OR "foetal demise" OR "menstrual age" OR "fetal age" OR "foetal age" OR "fetal growth" OR "foetal growth" OR "embryo growth" OR "fetal development" OR "foetal development" OR infant OR neonatal) AND TS=("foot length" OR "foot size" OR "foot measurement" OR "footlength" OR ("foot" AND anthropometr*) OR "foot measure")	147
August 22, 2018*	Popline*	foot length OR foot size OR foot measurement OR footlength OR foot anthropometry OR foot anthropometric OR foot measure (search active until September 1, 2019)	11
April 29, 2020	LILACS* (WHO Global Health Libraries)	tw:("foot length" OR "foot size" OR "foot measurement" OR "footlength" OR ("foot" AND anthropometr*) OR "foot measure") AND (instance:"ghl") AND (db:("LILACS" OR "IMSEAR" OR "WPRIM" OR "IMEMR" OR "AIM"))	63
August 22, 2018*	IMSEAR* (WHO Global Health Libraries)	tw:("foot length" OR "foot size" OR "foot measurement" OR "footlength" OR ("foot" AND anthropometr*) OR "foot measure") AND (instance:"ghl") AND (db:("LILACS" OR "IMSEAR" OR "WPRIM" OR "IMEMR" OR "AIM"))	30
August 22, 2018*	WPRIM* (WHO Global Health Libraries)	tw:("foot length" OR "foot size" OR "foot measurement" OR "footlength" OR ("foot" AND anthropometr*) OR "foot measure") AND (instance:"ghl") AND (db:("LILACS" OR "IMSEAR" OR "WPRIM" OR "IMEMR" OR "AIM"))	29
August 22, 2018*	IMEMR* (WHO Global Health Libraries)	tw:("foot length" OR "foot size" OR "foot measurement" OR "footlength" OR ("foot" AND anthropometr*) OR "foot measure") AND (instance:"ghl") AND (db:("LILACS" OR "IMSEAR" OR "WPRIM" OR "IMEMR" OR "AIM"))	20
August 22, 2018*	AIM* (WHO Global Health Libraries)	tw:("foot length" OR "foot size" OR "foot measurement" OR "footlength" OR ("foot" AND anthropometr*) OR "foot measure") AND (instance:"ghl") AND (db:("LILACS" OR "IMSEAR" OR "WPRIM" OR "IMEMR" OR "AIM"))	2

*These databases only reflect the search results from the initial searches in 2018 because they were not accessible when the final searches were completed in April 2020.

WebAppendix 4a. Methods: Conversion of Vertical Foot Length to Heel-Hallux Distance

The methods of foot length measurement varied slightly between studies (Table 1). All studies except one measured the heel-hallux distance. Mullany et al (2007) developed a special measuring board to measure the vertical distance.[3] In order to convert the vertical distance to a heel-hallux distance, we used the following equation (eFigure 1):

$$\textit{Heel hallux distance} = \textit{vertical distance} / \textit{cosign}\ (\alpha)$$

In order to estimate a typical α in a newborn foot, the vertical distance and heel-to-hallux distance was measured in a sample of 5 newborns. To obtain the average angle between the vertical and the heel-to-hallux sides of the right angle triangle that is formed, we used the following formula:

$$Angle\ (radians) = \cos^{-1}\left(\frac{vertical\ distance}{heel - to - hallux\ distance}\right)$$

The angle thus obtained was converted from radians to degrees. The conversion tables and data used are shown in WebAppendix 4b.

eFigure 1. Converting Vertical Foot Distance to Heel-Hallux Distance.
(b) shows the vertical distance and (c) shows the heel-hallux distance.

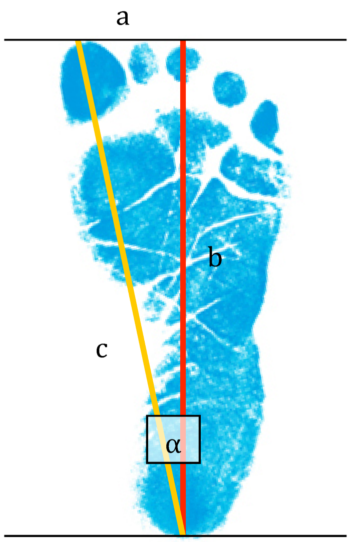

Folger LV, *et al. BMJ Global Health* 2020; 5:e002976. doi: 10.1136/bmjgh-2020-002976

WebAppendix 4b. Data from the Foot Length Angle Conversion

We measured the vertical distance and heel-to-hallux distance in 5 newborns. To obtain the angle between the vertical and the heel-to-hallux sides of a right angle triangle that is formed, we used the following formula:

$$Angle\ (radians) = \cos^{-1}\left(\frac{vertical\ distance}{heel - to - hallux\ distance}\right)$$

The angle thus obtained was converted from radians to degrees (Table 1).

Table 1. Foot Length Angle Derivation

Heel-Halux (cm)	Vertical (cm)	Angle (rad)	Angle (deg)
8	7.4	0.389760733	22.33165
8.6	8.3	0.264909197	15.17818
8.5	8.2	0.266472162	15.26773
7.4	7	0.330297355	18.92464
8	7.6	0.317560429	18.19487
	**Mean Angle**	**0.313799975**	**17.97941**

Using the mean angle (radians) obtained above, we converted the vertical foot length cutoffs used in Mullany et al, 2007[3] to heel-to-hallux foot length (Tables 2a and 2b).

Table 2a. Conversion of vertical foot length cutoffs for identifying <2500 gm neonates in Mullany et al, 2007. Data for sensitivity and specificity are from Table 2 in Mullany et al, 2007[3].

**Vertical Distance Foot Length Cutoff (cm)**	**Calculated Heel-Hallux Distance Cutoff (cm)**	**Heel-Halux Distance Cutoff (cm) (with rounding)**	**Sensitivity (%)** (From Mullany et al, 2007)	**Specificity (%)** (From Mullany et al, 2007)
6.8	7.148877092	7.1	36.9	96.6
6.9	7.254007638	7.3	47.1	94.2
7.0	7.359138183	7.4	57.4	90.0
7.1	7.464268729	7.5	73.1	80.4
7.2	7.569399274	7.6	81.2	69.6
7.3	7.67452982	7.7	88.7	56.4
7.4	7.779660365	7.8	94.5	43.7
7.5	7.88479091	7.9	97.4	32.7

Table 2b. Conversion of vertical foot length cutoffs for identifying <2000 gm neonates in Mullany et al, 2007. Data for sensitivity and specificity are from Table 3 in Mullany et al, 2007[3].

**Vertical Distance Foot Length Cutoff (cm)**	**Calculated Heel-Hallux Distance Cutoff (cm)**	**Heel-Hallux Distance Cutoff (cm)** (with rounding)	**Sensitivity (%)** (From Mullany et al, 2007)	**Specificity (%)** (From Mullany et al, 2007)
6.5	6.833485456	6.8	47.5	98.1
6.6	6.938616001	6.9	51.3	96.5
6.7	7.043746547	7.0	63.8	94.2
6.8	7.148877092	7.1	77.5	90.3
6.9	7.254007638	7.3	87.5	86.0
7.0	7.359138183	7.4	92.5	80.0
7.1	7.464268729	7.5	95.0	68.1
7.2	7.569399274	7.6	96.3	57.7
7.3	7.67452982	7.7	97.5	45.6

WebAppendix 5. Overall Study Table

Author	Year	Place (district/city, country)	Study Setting (NICU, hospital/tertiary care center, primary clinic, community)	Sample Size (n)	Sample Characteristics	Type of Data Available (Normative, Accuracy, Correlation, Reliability)
Ahmed[4]	2014	India (Karad)	Maternity ward, Krishna Hospital & Medical Research Centre	1028	582 males, 446 females	Diagnostic accuracy for <2500g; Correlation FL vs BW
Alia[5]	2011	Bangladesh (Dhaka)	Pediatrics & Obstetrics depts., Bangabandhu Sheikh Mujib Medical University	100	n=39 preterm; n=58 term; n=3 post-term; n=52 LBW	Correlation FL vs GA; Correlation FL vs BW
Ashish[6]	2015	Nepal (Kathmandu)	Tertiary, government-run, referral maternity hospital	811	51.3% male; 3.7% <2000 gm; 6.7% preterm	Diagnostic accuracy for preterm; Diagnostic accuracy for <2000g
Daga[7]	1988	India (Bombay, now Mumbai)	General hospitals, "cater to lower & lower-middle classes"	200	–	Correlation FL vs GA; Correlation FL vs BW; Reliability
Gavhane[8]	2016	India (Aurangabad)	Tertiary neonatal care unit, MGM Medical College teaching hospital	800	GA: 26-42 wks; 15.5% preterm; 25.4% LBW; 13.7% preterm AGA; 1.8% preterm SGA; 9.0% term SGA: 63.6% term AGA	Correlation FL vs GA; Correlation FL vs BW
Gidi[9]	2020	Ethiopia (Jimma)	Jimma University Medical Center, a tertiary referral hospital	1486	55% male; 10.2% preterm	Diagnostic accuracy for preterm; Diagnostic accuracy for <2500g; Correlation FL vs. GA; Correlation FL vs. BW
Gohil[10]	1991	India (Ahmedabad)	The Civil Hospital	353	n=37 preterm; n=105 term SGA; n=211 term AGA	Reliability
Gueye*[11]	2014	Senegal	–	251	n=108 BW <2500g	Diagnostic accuracy for <2500g; Correlation FL vs BW
Hadush[12]	2017	Ethiopia (Mekelle city, Tigray Region)	Ayder Referral hospital	422	50.24% male; BW range: 770-4760 gm; BW mean (SD): 2807 (692) gm; 18.18% preterm	Diagnostic accuracy for <2500g; Correlation FL vs BW
Hernandez[13]	1982	USA (Colorado)	Hospital	340	GA: 24-43 weeks	Correlation FL vs GA
Hirve[14]	1993	India (Pune district)	Community-based, 45 villages, rural area	89	43.84% LBW (<2500 gm)	Reliability
James[15]	1979	England (Manchester)	St. Mary's Hospital	123	GA: 26-42 wks; 54% AGA (n=66); 46% SGA (n=57); 19% preterm (n=23)	Correlation FL vs BW; Correlation FL vs BW; Reliability
Kulkarni & Rajendran[16]	1992	India (Karnataka)	–	817	GA: 26-42 wks	Normative data
Lee[a17]	2016	Bangladesh (Sylhet)	Community-based	710	GA: 29.6–44.0 wks; 32.4% SGA; 8.3% preterm	Diagnostic accuracy for preterm; Correlation FL vs GA
Lee[a18]	2017	Bangladesh (Sylhet)	Community-based	1858	LBW <2500 gm: 21.4%; VLBW <2000 gm: 2.2%	Diagnostic accuracy for <2500g; Diagnostic accuracy for <2000g
Madhulika[19]	1989	India (Jaipur)	–	1000	GA: 28-42 wks	Normative data; Correlation FL vs GA
Marchant[20]	2010	Tanzania (Lindi and Mtwara regions)	Mtwara Regional hospital (known as "Ligula Hospital")	529	Mean GA (SD): 39.5 (2.4) wks; Mean BW (SD): 2.9 (0.4) kg; 8% preterm; 15% LBW	Diagnostic accuracy for preterm; Diagnostic accuracy for <2500g
Marchant[21]	2014	Tanzania (Mtwara region)	Community-based	144	–	Reliability
Mathur[22]	1984	India (Udaipur)	Hospital	300	GA: 28-42 wks; 55.7% male; Term >2500 gm: 29.3 (n=88); Term LBW: 32.3% (n=97); Preterm: 38.3% (n=115)	Normative data; Correlation FL vs GA; Correlation FL vs BW
Merlob[23]	1984	Israel	–	198	GA: 27-41 wks; Male: n=108; Female: n= 95;	Normative data
Modibbo[24]	2013	Nigeria (Kano State)	Murtala Muhammad Specialist Hospital Kano (a referral centre)	551	BW range: 1.50-5.50 kg; Mean BW (SD): 3.08 (0.55) kg;	Correlation FL vs BW
Mukherjee[25]	2013	India (Kolkata)	Tertiary care hospital	351	Mean GA (SD): 36.37 (3.6) wks; Mean BW (SD): 2090 (810) gm; 48.1% preterm; 51.8% LBW	Diagnostic accuracy for preterm; Diagnostic accuracy for <2500g; Correlation FL vs GA; Correlation FL vs BW; Reliability
Mullany[3]	2007	Nepal (Sarlahi district)	Community-based	1640	50.1% male; 28.6% LBW; 4.9% <2000 gm	Diagnostic accuracy for <2500g; Diagnostic accuracy for <2000g; Correlation FL vs BW; Reliability

Nabiwemba[b 26]	2013a	Uganda (Iganga)	Iganga General Hospital	711	54% male; 4% preterm; BW range: 1370-5350 gm; 12% LBW	Diagnostic accuracy for preterm; Diagnostic accuracy for <2500g
Nabiwemba[b 27]	2013b	Uganda (Iganga)	Iganga General Hospital	706	BW range: 1370-5350 gm; 12% LBW	Correlation FL vs BW; Reliability
Naqvi[28]	1986	USA (Texas)	Hospital nursery	132	GA: 20-41 wks; Male: n=71; Female: n=61	Correlation FL vs GA
Otupiri[29]	2014	Ghana (Kumasi)	2 public hospitals (Komfo Anokye Teaching Hospital & Suntreso Government Hospital)	973	20.7% preterm; 21.7% LBW <2500 gm; 2.9% <1500 gm	Diagnostic accuracy for <2500g; Correlation FL vs BW
Paulsen[30]	2019	Tanzania (Korogwe, Handeni)	Community-based	376	51.3% female; 10.4% LBW <2500 gm; 18.4% SGA; 4.5% preterm	Diagnostic accuracy for preterm; Diagnostic accuracy for <2500g; Correlation FL vs. GA; Correlation FL vs. BW
Pratinidhi[31]	2017	India (Karad, Maharashtra)	Krishna Hospital and Medical Research Centre	645	–	Diagnostic accuracy for preterm; Diagnostic accuracy for <2500g; Diagnostic accuracy for <2000g; Correlation FL vs GA; Correlation FL vs BW
Rakkappan & Kuppusaamy[32]	2016	India (South Tamil Nadu)	Government Rural Medical College Hospital	1000	GA: 26-42 wks; 53.7% male, 48.3% female; 18.6% preterm; 85.1% AGA, 14.3% SGA;	Normative data
Roy[33]	2019	India (Mangalore)	Tertiary hospital affiliated to Kasturba Medical College	320	49.7% male; 17.5% preterm	Diagnostic accuracy for preterm; Correlation FL vs. GA
Rustagi[34]	2012	India (Delhi)	Community-based, urban	283	52.2% male; BW range: 1500-4200 gm; Mean BW (SD): 2726 (484) gm	Diagnostic accuracy for <2500g; Correlation FL vs BW
Sateesha[35]	2015	India (Bangalore)	Tertiary neonatal unit, teaching hospital, referral intensive care unit	312	GA: 28-41 wks; BW: 800-4500 gm; 40.7% preterm	Correlation FL vs GA; Correlation FL vs BW
Shah[36]	2005	Nepal	Tertiary hospital	1000	GA: 26-44 wks; 12.6% LBW	Correlation FL vs BW
Singhal[37]	2014	India (Jaipur)	Rural tertiary care hospital	1000	GA: 28-42 wks	Normative data; Diagnostic accuracy for preterm; Correlation FL vs GA
Srinivasa[38]	2017	India (Bengaluru)	KIMS Hospital and Research Center, Bengaluru, India	500	GA: 27-42 wks; 16.8% preterm; 15.2% SGA	Diagnostic accuracy for preterm; Diagnostic accuracy for <2500g; Correlation FL vs GA; Correlation FL vs BW
Srivastava[39]	2015	India (Delhi & Indore)	Tertiary hospitals	254	GA: 27-42 wks	Normative data; Correlation FL vs GA; Correlation FL vs BW
Thawani[40]	2013	India (Delhi)	Neonatology division, University College of Medical Sciences and GTB Hospital	1000	GA: 25-42 weeks; 37.3% preterm; BW range 685-4165 gm; BW mean (SD): 2395 (597) gm; AGA: n=850; SGA: n=142	Correlation FL vs GA; Correlation FL vs BW
Thi[41]	2015	Vietnam (Hoa Binh Province)	Maternal and Neonatal Departments, Hoa Binh Province General Hospital	485	Ethnic minority newborns (Tay, Dao, Nung, H'Mong, Muong, or San Chi); GA: 30-42 wks; BW: 1007-4500 gm; Mean BW (SD): 2489 (58) gm; 51% LBW; 47% preterm; 49% female	Diagnostic accuracy for preterm; Diagnostic accuracy for <2500g
Usher[42]	1969	Canada (Montreal)	Tertiary hospital	300	GA: 25-44 wks; 145 male, 155 female	Normative data
Vocel*[43]	1978	Czechoslovakia	–	288	GA: 29-43 wks; 136 female, 152 male	Normative data

Abbreviations: GA, gestational age; BW, birthweight; LBW, low birthweight <2500 gm; VLBW, very low birthweight <2000 gm; FL, foot length; SGA, small-for-gestational age; AGA, appropriate size for gestational age
(–) symbol indicates that data is not available for that paper.
*Translated
[a] The two studies by Lee et al (2016 and 2017) use the same cohort, but Lee (2016) uses a smaller subsample of the overall cohort.
[b] The two papers by Nabiwemba et al, both published in 2013, use the same cohort but conduct slightly different analyses, and one uses a slightly smaller proportion of the full sample (n=706 vs n=711); although Nabiwemba (2013b) reports some diagnostic accuracy data, we chose to report only the diagnostic accuracy data from the 2013a paper for consistency, since the populations are nearly identical.

WebAppendix 6. QUADAS-2 Summary

Overall study quality scores on the 4 domains measured by QUADAS-2 (Quality Assessment of Diagnostic Accuracy Studies-2, Whiting et al. 2011) for all included studies with diagnostic accuracy data, e.g. sensitivity, specificity, area under the curve (n=18*). Individual study level QUADAS-2 data is available upon request from the corresponding author.

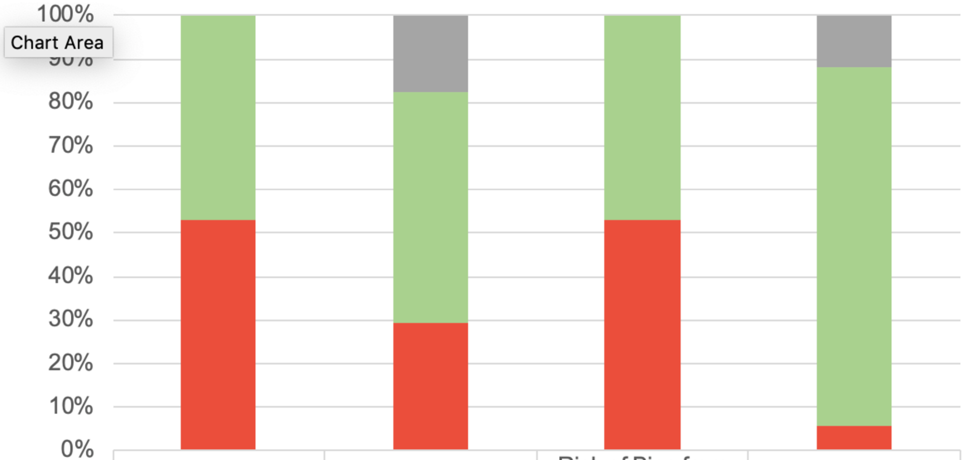

	Risk of Bias from Patient Selection	Risk of Bias from Test Method	Risk of Bias from Reference Standard	Risk of Bias from Flow and Timing
Unclear	0	3	0	2
Low	8	9	8	14
High	9	5	9	1

High Low Unclear

*These data summarize 18 of the 19 different studies in this review that reported on diagnostic accuracy because 2 studies reported on one cohort in two separate reports[17 18] and thus were scored only once based on Lee et al 2016[17].

WebAppendix 7. Neonatal Foot Length Measurement Methods

7a. Measurement of heel-to-hallux foot length using a hard plastic ruler. Photograph from the Lee et al. (2016[17] and 2017[18]) cohort, reproduced with permission.

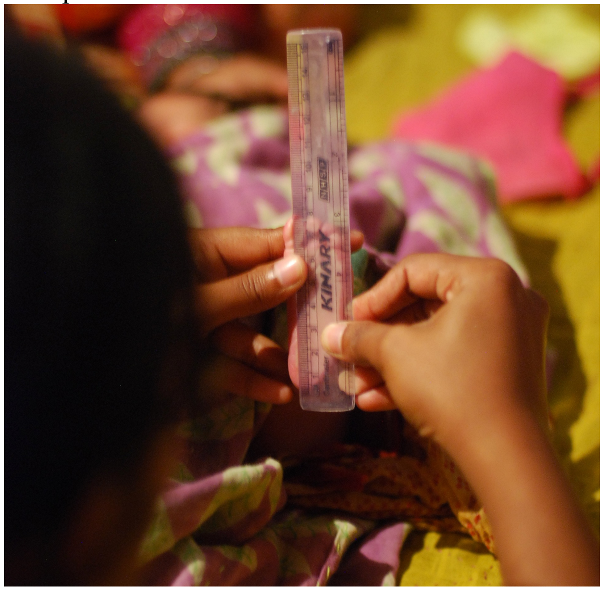

7b. Foot length measuring box/board. This was the device used in Mullany et al. (2007[3]), and picture is from Hodgins et al. (2020[44], preprint).

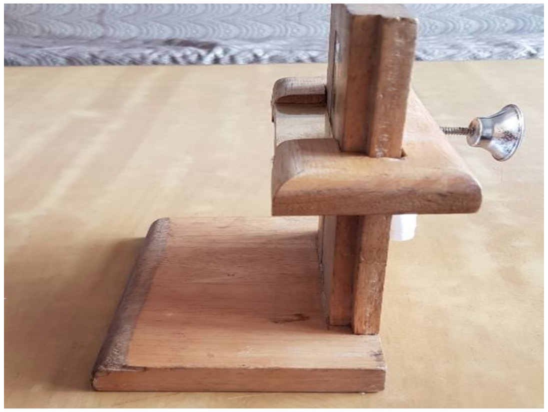

WebAppendix 8a. Correlation between Neonatal Foot Length and Gestational Age, by Reference Standard Gestational Age

Author (year)	Study Setting	Reference Standard GA Measurement	Correlation Coefficient	Type of Correlation Coefficient
**ULTRASOUND**				
Lee (2016)[17]	Community-based, Sylhet, Bangladesh	US	0.0931	Pearson's
Paulsen (2019)[30]	Community-based, Korogwe and Handeni, Tanzania	US	0.37	Spearman
**LAST MENSTRUAL PERIOD (LMP)**				
Mathur (1984)[22]	Hospital, Udaipur, India	LMP	0.98	–
Pratinidhi (2017)[31]	Krishna Hospital and Medical Research Centre, Karad, Maharashtra, India	LMP	0.63	–
**CLINICAL EXAM OR UNKNOWN REFERENCE STANDARD**				
Madhulika (1989)[19]	Jaipur, India	LMP & Dubowitz score	0.9399	–
Singhal (2014)[37]	Rural tertiary care hospital, Jaipur, India	LMP & "Extended" NBS	0.934	–
Hernandez (1982)[a 13]	Hospital, Colorado, USA	LMP & infant physical exam	0.982[a]	–
Naqvi (1986)[28]	Hospital nursery, Texas, USA	Ballard	0.9	–
Gavhane (2016)[b 8]	MGM Medical College tertiary hospital, Aurangabad, India	"Modified" Ballard Score	Preterm SGA: 0.75; Preterm AGA: 0.81; Term SGA: 0.48; Term AGA: 0.44	–
Mukherjee (2013)[25]	Tertiary care hospital, Kolkata, India	NBS	0.869	Pearson's
Roy (2019)[33]	Tertiary care hospital, Mangalore, India	NBS	Preterm: 0.274; Term: 0.088	–
Sateesha (2015)[35]	Tertiary neonatal unit, Bangalore, India	–	Preterm SGA: 0.598; Preterm AGA: 0.860; Term SGA: -0.158; Term AGA 0.371; Term LGA: 0.137	Pearson's
Srinivasa (2017)[38]	KIMS Hospital and Research Center, Bengaluru, India	NBS	0.876	–
Srivastava (2015)[39]	Tertiary hospitals in Delhi & Indore, India	NBS	0.99	–
Thawani (2013)[40]	University College of Medical Sciences and GTB Hospital, Delhi, India	NBS	0.43	Pearson's
Alia (2011)[5]	Bangabandhu Sheikh Mujib Medical University (BSMMU), Dhaka, Bangladesh	–	0.853	Pearson's
Daga (1988)[7]	General hospitals, Bombay (now Mumbai), India	–	0.711	–

Abbreviations: US/BOE, ultrasound or best obstetric estimate; LMP, last menstrual period; HIC, high-income countries (by World Bank definition); LMIC, low-and-middle-income countries; CI, confidence interval; SGA, small-for-gestational age; AGA, appropriate size for gestational age; LBW, low birthweight; GA, gestational age
(–) symbol indicates that data is not available for that paper.
[a] Hernandez (1982) only reported a correlation coefficient for infants <34 weeks gestational age; n for <24 wks is not stated in the paper.
[b] Gavhane (2016) included a full size and age range population, but data was analyzed in separate subgroups based on size-for-gestational-age & authors only reported the correlation coefficients with GA for 4 of 8 subgroups

Web Appendix 8b. Correlation between Neonatal Foot Length and Birthweight, by Region

Author (year)	Study Setting	Reference Standard for Birthweight Measurement	Correlation Coefficient (r)	Type of Correlation Coefficient
**ASIA**				
Ahmed (2014)[4]	Krishna Hospital and Medical Research Centre, Karad, India	Nude weight of the baby was taken in an electronic weighing machine, with an accuracy of ± 1 gram	Males: 0.461; Females: 0.505	–
Alia (2011)[5]	Bangabandhu Sheikh Mujib Medical University (BSMMU), Dhaka, Bangladesh	–	0.951	Pearson’s
Daga (1988)[7]	General hospitals, Mumbai (formerly Bombay), India	–	0.786	–
Gavhane (2016)[a 8]	MGM Medical College tertiary hospital, Aurangabad, India	Electronic weighing scale with accuracy of ± 5 grams	Term AGA: 0.495[a] (n=509)	–
Mathur (1984)[22]	Hospital, Udaipur, India	“beam type machine”, accuracy to 20 grams	Full term >2500g: 0.83; Term LBW: 0.89; Preterm: 0.91	–
Mukherjee (2013)[25]	Tertiary care hospital, Kolkata, India	Digital Salter scale	Preterm infants: 0.973; Term infants: 0.96	Pearson’s
Mullany (2007)[3]	Community-based, Sarlahi district, Nepal	Digital scale (SECA Digital Scale Model 727); precision of 2 grams	0.68	–
Pratinidhi (2017)[31]	Krishna Hospital and Medical Research Centre, Karad, Maharashtra, India	Nude weight taken using standard electronic weighing machine (Vijay Digital System, 24B Technology)	0.75	–
Rustagi (2012)[34]	Community-based, urban, Delhi, India	Weighed naked or in minimal clothing using a Salter’s spring-type scale to the nearest 50 grams; scale zeroed before each measurement	0.213	–
Sateesha (2015)[35]	Tertiary neonatal unit, Bangalore, India	–	Preterm SGA: 0.583; Preterm AGA: 0.714; Term SGA: 0.122; Term AGA: 0.226; Term LGA: 0.522	Pearson’s
Shah (2005)[36]	Tertiary hospital, Nepal	–	0.92	–
Srinivasa (2017)[38]	KIMS Hospital and Research Center, Bengaluru, India	–	0.9	–
Srivastava (2015)[39]	Tertiary hospitals in Delhi & Indore, India	Weighed naked on weighing machine (BRAUN Company) to nearest 50 gm	0.94	–
Thawani (2013)[40]	University College of Medical Sciences and GTB Hospital, Delhi, India	Weighed nude on digital electronic scale (Goldtech, Merino International) to the nearest 5 gm	0.508	Pearson’s
**AFRICA**				
Gueye (2014)[11]	Senegal	–	0.927	Pearson’s
Hadush (2017)[12]	Ayder Referral hospital, Mekelle city, Tigray region, north Ethiopia	Weighed naked on a digital weighing scale; scale calibrated before every measurement using a material of standard weight 1000 gm	0.746	Pearson’s

Modibbo (2013)[24]	Murtala Muhammad Specialist Hospital Kano (a referral centre), Kano State, Nigeria	Digital weighing scale (Model: ACS – 20; Country: China)	0.657	–
Nabiwemba (2013b)[27]	Iganga General Hospital, Iganga, Eastern Uganda	Digital Salter scale to the nearest 10 grams; scale calibrated before each use using a 1000 gm bottle	0.76	Pearson's
Otupiri (2014)[29]	Komfo Anokye Teaching Hospital and Suntreso Government Hospital, Kumasi, Ghana	Model 180 Salter weighing scale, calibrated with a bottle weighing 1000 gm; weighed to the nearest 100 gm	$\geq$2500 gm: 0.23; <2500 gm: 0.53; <1500 gm: 0.44	Pearson's pairwise correlation method
Paulsen (2019)[30]	Community-based, Korogwe and Handeni, Tanzania	Nude weight taken using a digital weighing scale (M107600, ADE, Germany) and noted to nearest 5g	0.66	Pearson's
**EUROPE**				
James (1979)[15]	St. Mary's Hospital, Manchester, England	"known birthweights"	AGA: 0.89; SGA: 0.91; Preterm: 0.95	–

Abbreviations: SGA, small-for-gestational age; AGA, appropriate size for gestational age; LBW, low birthweight; GA, gestational age; BW, birthweight
(–) symbol indicates that data is not available for that paper.
[a] Study included a full size and age range population, but data was analyzed in separate subgroups based on size-for-gestational-age & only reported the correlation with birth weight for the one subgroup with the highest correlation (term AGA)

WebAppendix 9. Areas Under the Curve for Foot Length to Identify Low Birthweight Infants

Author (year)	Study Setting	Reference Standard Birthweight	AUC (95% CI)
**LOW BIRTHWEIGHT (<2500 gm)**			
Gidi (2020)[9]	Tertiary hospital, Jimma, Ethiopia	Digital Salter scale to the nearest 10 g	0.85 (0.83-0.87)
Hadush (2017)[12]	Tertiary hospital, Mekelle city, Ethiopia	Weighed naked on digital scale; scale calibrated before every measurement	0.897 (0.861 – 0.934)
Mullany (2007)[3]	Community-based, Sarlahi district, Nepal	Digital scale, calibrated before each measurement	0.84
Nabiwemba (2013a)[26]	Tertiary hospital, Iganga, Uganda	Digital Salter scale (resolution 10 gm); calibrated before each measurement	0.97
Otupiri (2014)[29]	Tertiary hospitals, Kumasi, Ghana	Model 180 Salter weighing scale to nearest 100 gm; was calibrated	0.74
Paulsen (2019)[30]	Community-based, Korogwe and Handeni, Tanzania	Nude weight taken using a digital weighing scale (M107600, ADE, Germany) and noted to nearest 5g	0.8528
Thi (2015)[41]	Tertiary hospital, Hoa Binh Province, Vietnam	Salter WS034 digital weighing scale to nearest 10 gm; calibrated periodically	0.94 (0.92 – 0.96)
**VERY LOW BIRTHWEIGHT (<2000 gm)**			
Ashish (2015)[6]	Tertiary Hospital, Kathmandu, Nepal	Plastic pan scale with 50 gm unit of measurement (Narang Medical limited WS590); calibrated at zero before each measurement	0.878
Mullany (2007)[3]	Community-based, Sarlahi district, Nepal	Digital scale, calibrated before each measurement	0.93

Abbreviations: AUC, area under the curve; CI, confidence interval

Folger LV, *et al. BMJ Global Health* 2020; 5:e002976. doi: 10.1136/bmjgh-2020-002976

WebAppendix 10a-c. Diagnostic Accuracy of Foot Length to Identify Low Birthweight (<2500 gm) and Very Low Birthweight (<2000 gm) Infants, by World Region. (a) Foot Length to identify infants <2500 gm in Asia; (b) Foot Length to identify infants <2500 gm in Africa; (c) Foot length to identify infants <2000 gm in Asia.

10a. Diagnostic accuracy of foot length to identify infants <2500 gm in Asia

**Foot Length Cutoff, cm** (as reported in paper)	**Author (year)**	**N**	**% LBW**	**Sensitivity (%) (95% CI)**	**Specificity (%)(95% CI)**	**PPV (%) (95% CI)**	**NPV (%) (95% CI)**
7.0	Ahmed (2014)[4]	446 (females only)	–	38.7	87.5	66.33	69.27
≤7.0	Rustagi (2012)[34]	283	–	58.1	83.0	31.5	–
<7.1[a]	Mullany (2007)[3]	1640	28.6	36.9	96.6	81.2	79.3
≤7.2	Lee[b] (2017)[18]	1858	21.4	33.6	81.0	32.4	81.8
<7.3[a]	Mullany (2007)	1640	28.6	47.1	94.2	76.5	81.6
≤7.3	Pratidinhi (2017)[31]	645	32.9[c]	79.7 (73.7-84.9)	70.0 (65.4-74.2)	–	–
≤7.3	Lee (2017)	1858	21.4	38.4	77.8	31.9	82.3
<7.4[a]	Mullany (2007)	1640	28.6	57.4	90.0	69.7	84.1
≤7.4[d]	Srinivasa (2017)[38]	500	–	97.03 (91.6-99.4)	81.95 (77.8-85.6)	–	–
<7.5[d]	Rustagi (2012)	283	–	57.5	84.6	40.3	–
≤7.4	Lee (2017)	1858	21.4	44.4	71.4	29.6	82.6
≤7.4	Thi (2015)[41]	485	51	85 (79-89)	86 (81-90)	86 (81-90)	84 (79-89)
<7.5[a]	Mullany (2007)	1640	28.6	73.1	80.4	59.9	88.2
≤7.5	Lee (2017)	1858	21.4	56.6	63.0	29.3	84.2
<7.6[a]	Mullany (2007)	1640	28.6	81.2	69.6	51.7	90.3
≤7.6	Lee (2017)	1858	21.4	69.2	53.8	28.9	86.6
<7.7[a]	Mullany (2007)	1640	28.6	88.7	56.4	44.9	92.6
≤7.7	Lee (2017)	1858	21.4	74.5	48.1	28.0	87.4
<7.8[a]	Mullany (2007)	1640	28.6	94.5	43.7	40.2	95.2
"less than 7.85"	Mukherjee (2013)[25]	351	51.8	100.0	95.3	–	–
7.8	Ahmed (2014)	582 (males only)	–	90.9	21.2	33.3	84.31
≤7.8	Lee (2017)	1858	21.4	81.6	35.2	25.5	87.6
<7.9[a]	Mullany (2007)	1640	28.6	97.4	32.7	36.7	97.0
<8.0	Rustagi (2012)	283	–	41.0	86.3	56.1	–

Abbreviations: LBW, low birthweight; PPV, positive predictive value; NPV, negative predictive value
(–) symbol indicates that data is not available for that paper.
[a] Mullany cutoffs reported in the original paper were converted from vertical foot length distance to heel-halux distance for pooling. Details are in WebAppendix 4a-b.
[b] Lee (2017) diagnostic accuracy data for <2500 gm infants are unpublished; data was obtained from the author.
[c] Percent LBW calculated by authors (LVF, PP, ACC) from data reported in original paper.
[d] Not included in pooled analysis for ≤7.4 cm cutoff because % LBW data was missing.

10b. Diagnostic accuracy of oot length to identify infants <2500 gm in Africa

Foot Length Cutoff, cm (as reported in paper)	Author (year)	N	% LBW	Sensitivity (%) (95% CI)	Specificity (%) (95% CI)	PPV (%) (95% CI)	NPV (%) (95% CI)
<7.0	Gueye (2014)[11]	251	43	44.44	100	100	70.44
7.2	Nabiwemba (2013a)[26]	711	12	38.3	97.9	71.7	92.1
7.3	Nabiwemba (2013a)	711	12	49.4	96.3	64.6	93.3
7.35	Hadush (2017)[12]	422	27	72.8	91.6	–	–
≤7.4	Otupiri (2014)[29]	973	21.7	–	–	63.0 (54.2-71.1)	85.0 (82.4-87.3)
7.4	Nabiwemba (2013a)	711	12	54.1	95.3	61	93.9
7.5	Nabiwemba (2013a)	711	12	58.8	93.7	55.9	94.4
7.6	Nabiwemba (2013a)	711	12	85	81	38.3	97.5
≤7.6	Paulsen (2019)[30]	376	10.4	79 (64–91)	75 (70–79)	0.27 (0.19–0.36)	0.97 (0.94–0.99)
≤7.7	Gidi (2020)[9]	1389	14.6	84.2 (78.4 to 88.9)	73.9 (71.3 to 76.4)	35.4 (31.1 to 39.9)	96.5 (95.1 to 97.6)
7.7	Nabiwemba (2013a)	711	12	87.1	78.6	35.6	97.8
≤7.7	Paulsen (2019)	376	10.4	82 (66–92)	67 (61–72)	0.22 (0.16–0.30)	0.97 (0.94–0.99)
7.8	Nabiwemba (2013a)	711	12	87.1	78.6	35.6	97.8
7.9[a]	Nabiwemba (2013a)[b]	711	12	92.9	72.4	31.4	98.7
<8.0	Gueye (2014)	251	43	95.37	81.11	79.23	95.87
<8.0	Marchant (2010)[20]	529	15	87	60	24	96
8.0	Nabiwemba (2013a)	711	12	94.1	71.2	30.9	98.9
8.1	Nabiwemba (2013a)	711	12	97.7	44.4	19.3	99.3
8.2	Nabiwemba (2013a)	711	12	98.9	28.5	15.9	99.4

Abbreviations: CI, confidence interval; PPV, positive predictive value; NPV, negative predictive value; AUC, area under the curve
(–) symbol indicates that data is not available for that paper.
[a] It is not stated in the paper if the cutoffs are < or ≤ ; for the purposes of pooling, we used ≤7.9cm.
[b] Of note, 2 of the papers that reported diagnostic accuracy in Uganda appeared to be from the same sample population[26,27]; only one (Nabiwemba 2013a) had data that could be included in the meta-analysis of studies identifying <2500 gm infants in Africa[26]

10c. Diagnostic accuracy of foot length to identify infants <2000 gm in Asia

**Foot Length Cutoff, cm** (as reported in paper)	**Author (year)**	**N**	**% VLBW (<2000g)**	**Sensitivity (%) (95% CI)**	**Specificity (%) (95% CI)**	**PPV (%)**	**NPV (%)**
<6.8[a]	Mullany (2007)[3]	1640	4.9	47.5	98.1	56.7	97.3
≤6.8	Ashish KC (2015)[6]	811	3.7[b]	24.1	98.9	73.0	97.0
≤6.8	Lee[c] (2017)[18]	1858	2.2	48.7	91.9	11.4	98.8
≤6.8	Pratinidhi (2017)[31]	645	12.9[d]	92.8 (84.9-97.3)	92.3 (89.9-94.4)	–	–
<6.9	Mullany (2007)	1640	4.9	51.3	96.5	42.7	97.5
≤6.9	Ashish KC (2015)	811	3.7	31.0	98.2	68.0	97.0
≤6.9	Lee (2017)	1858	2.2	48.7	90.9	10.3	98.8
<7.0	Mullany (2007)	1640	4.9	63.8	94.2	35.9	98.1
≤7.0	Ashish KC (2015)	811	3.7	34.5	97.6	60.9	97.6
≤7.0	Lee (2017)	1858	2.2	56.4	86.0	8.0	98.9
<7.1	Mullany (2007)	1640	4.9	77.5	90.3	29.1	98.7
≤7.1	Ashish KC (2015)	811	3.7	72.4	90.8	28.9	99.0
≤7.1	Lee (2017)	1858	2.2	64.1	83.9	7.9	99.1
≤7.2	Ashish KC (2015)	811	3.7	75.9	90.3	27.8	99.1
≤7.2	Lee (2017)	1858	2.2	71.8	79.0	6.8	99.2
<7.3	Mullany (2007)	1640	4.9	87.5	86.0	24.2	99.3
≤7.3	Ashish KC (2015)	811	3.7	75.9	88.6	24.7	99.1
≤7.3	Lee (2017)	1858	2.2	71.8	75.3	5.9	99.2
<7.4	Mullany (2007)	1640	4.9	92.5	80.0	19.2	99.5
≤7.4	Ashish KC (2015)	811	3.7	79.3	86.8	22.1	99.2
≤7.4	Lee (2017)	1858	2.2	74.4	68.9	4.9	99.2
<7.5	Mullany (2007)	1640	4.9	95.0	68.1	13.3	99.6
≤7.5	Ashish KC (2015)	811	3.7	82.8	85.2	20.2	99.4
≤7.5	Lee (2017)	1858	2.2	82.1	59.7	4.2	99.4
<7.6	Mullany (2007)	1640	4.9	96.3	57.7	10.4	99.7

Abbreviations: CI: confidence interval, PPV: positive predictive value, NPV: negative predictive value
(–) symbol indicates that data is not available for that paper.
[a] Mullany cutoffs reported in the original paper were converted from vertical foot length distance to heel-halux distance for pooling. Details are in WebAppendix 4a-b.
[b] Email communications with author determined ≤ cutoff for Ashish.
[c] Lee (2017) data for most cutoffs are unpublished; data was obtained from the author.
[d] Percent calculated by authors (LVF, PP, ACC) from data reported in original paper.

Folger LV, *et al. BMJ Global Health* 2020; 5:e002976. doi: 10.1136/bmjgh-2020-002976

WebAppendix 11. Inter- and Intra-rater Reliability of Neonatal Foot Length Measurements

Author (year)	Study Setting	Sample Size (n) for Comparison	Foot Length Measurement Tool & Method	Types of Assessor(s)	Kappa Statistic (inter-rater)	Other Inter-rater Reliability	Intra-rater Reliability
Daga (1988)[7]	General hospitals, Mumbai (formerly Bombay), India	5 preterm & 5 term infants	Footprint on plain paper; maximum distance between heel & big toe	–	–	77.7% agreement	90% agreement
Gohil (1991)[10]	Civil Hospital, Ahmedabad, India	n=1 preterm infant; n=1 term SGA; n=1 term AGA	Sliding gauge with precision 0.05 cm; distance from heel to big toe	–	–	10 different observers measured FL once on same baby; preterm: 1.23% variation; term SGA: 1.46%; term AGA: 1.6%	10 measurements on 1 baby in each category by the same observer; preterm: 1.2% variation; term SGA: 1.4%; term AGA: 1.56%
Hirve (1993)[14]	Community-based, 45 villages, rural area of Pune District, India	89	Paper tape with 3 color zones that correlated to foot size & care instructions; distance from heel to tip of longest toe	Social worker vs. caretaker of the neonate (often mother)	κ = 0.82	Intra-class correlation coefficient: 0.896	–
James (1979)[15]	St Mary's Hospital, Manchester, England	1	Perspex neonatal foot length gauge, precision to 0.5 mm; heel to tip of big toe	Inter-rater: medical and nursing staff on the special care baby unit; Intra rater: 1 researcher	–	10 different observers measured FL once on same baby; coefficient of variation: 1.46%	10 measurements on 1 baby by the same observer; coefficient of variation: 1.05%
Marchant (2014)[21]	Community-based, Southern Tanzania	142	(1) Counseling card to classify FL as very short (<7.0 cm), short (7-7.9 cm), or not-short ($\geq$8cm); (2) plastic ruler	Researcher vs. community volunteer	κ = 0.53 (95% CI: 0.40-0.66); for classification of FL $\geq$8 cm or <8 cm using counseling card	Mean difference (researcher – volunteer, using plastic ruler): 0.26 cm (SD 0.27; 95% CI 0.22 – 0.30), positive skew; Bland-Altman: no evidence of bias	–
Mukherjee (2013)[25]	Tertiary care hospital, Kolkata, India	351	Stiff plastic transparent ruler; distance from heel to tip of great toe	4 different physicians measured each infant	κ = 0.81	–	–
Mullany (2007)[3]	Community based, Sarlahi district, Nepal	1640	Sliding level on a vertical ruler (precision 0.1 cm), heel against stabilizing board; vertical FL (heel to tip of toe)	A non-medical field worker made 3 measurements on each infant	–	–	Within-infant range of measures was ≤0.2 cm in 98.4% of infants
Nabiwemba (2013b)[27]	Iganga General Hospital, Iganga, Eastern Uganda	24	Hard transparent plastic ruler, precision 0.1 cm; heel to tip of big toe	Midwife vs. supervisor	κ = 0.30	–	–

Abbreviations: LMP, last menstrual period; BOE, best obstetric estimate; US, ultrasound; CI, confidence interval; LOA, limits of agreement; GA, gestational age; FL, foot length

WebAppendix References

1. Moher D, Liberati A, Tetzlaff J, Altman DG, Group P. Preferred reporting items for systematic reviews and meta-analyses: the PRISMA statement. *BMJ (Clinical research ed)* 2009; **339**: b2535.
2. Macaskill P, Gatsonis C, Deeks J, Harbord R, Y T. Chapter 10: Analysing and Presenting Results. In: Deeks JJ, Bossuyt PM, C G, eds. Cochrane Handbook for Systematic Reviews of Diagnostic Test Accuracy Version 10: The Cochrane Collaboration; 2010.
3. Mullany LC, Darmstadt GL, Khatry SK, Leclerq SC, Tielsch JM. Relationship between the surrogate anthropometric measures, foot length and chest circumference and birth weight among newborns of Sarlahi, Nepal. *Eur J Clin Nutr* 2007; **61**(1): 40-6.
4. Ahmed M, Colaco SM, Ali MA, Ahmad Z. Birth Weight Status of Newborn and Its Relationship With Other Anthropometric Parameters. *International Journal of Medical and Health Sciences* 2014; **3**(1).
5. Alia RA, Mannan MA, Fatema K, Begum F, Siddique R. Correlation of birth weight with other anthropometric variables in detection of low birth weight (LBW) babies. *J Dhaka National Med Coll Hos* 2011; **17**(1): 29-32.
6. Ashish KC, Nelin V, Vitrakoti R, Aryal S, Malqvist M. Validation of the foot length measure as an alternative tool to identify low birth weight and preterm babies in a low-resource setting like Nepal: a cross-sectional study. *BMC Pediatr* 2015; **15**: 43.
7. Daga SR, Daga AS, Patole S, Kadam S, Mukadam Y. Foot length measurement from foot print for identifying a newborn at risk. *Journal of tropical pediatrics* 1988; **34**(1): 16-9.
8. Gavhane S, Kale A, Golawankar A, Sangle A. Correlation of foot length and gestational maturity in neonates. *International Journal of Contemporary Pediatrics* 2016; **3**(3): 705-8.
9. Gidi NW, Berhane M, Girma T, et al. Anthropometric measures that identify premature and low birth weight newborns in Ethiopia: a cross-sectional study with community follow-up. *Archives of disease in childhood* 2020; **105**(4): 326-31.
10. Gohil JR, Sosi M, Vani SN, Desai AB. Footlength Measurement in the Neonate. *Indian journal of pediatrics* 1991; **58**(5): 675-7.
11. Gueye M, Sylla A, Ramatoulaye Diagne/Gueye N, Moctar Faye P, Ali Said Mbae A, Ndiaye O. Screening of low-birthweight babies with foot length measurement. *Archives de Pediatrie* 2014; **21**.
12. Hadush MY, Berhe AH, Medhanyie AA. Foot length, chest and head circumference measurements in detection of Low birth weight neonates in Mekelle, Ethiopia: a hospital based cross sectional study. *BMC Pediatr* 2017; **17**(1): 111.
13. Hernandez JA, Lazarte RA, Pisanos DE, Butterfield LJ. Foot Length and Gestational Age (abstract only). *Clinical Research* 1982; **30**(1): A121.
14. Hirve SS, Ganatra BR. Foot tape measure for identification of low birth weight newborns. *Indian pediatrics* 1993; **30**(1): 25-9.
15. James DK, Dryburgh EH, Chiswick ML. Foot length--a new and potentially useful measurement in the neonate. *Archives of disease in childhood* 1979; **54**(3): 226-30.
16. Kulkarni ML, Rajendran NK. Values for foot length in newborns. *Indian pediatrics* 1992; **29**(4): 507-9.
17. Lee AC, Mullany LC, Ladhani K, et al. Validity of Newborn Clinical Assessment to Determine Gestational Age in Bangladesh. *Pediatrics* 2016; **138**(1).
18. Lee AC, Ladhani K, Rahman M, et al. Foot Length and Surrogate Neonatal Anthropometrics to Identify Preterm and Low Birth Weight Infants in Rural Bangladesh. Pediatric Academic Societies (PAS) Annual Meeting; 2017 May 6-9, 2017; San Francisco, CA; 2017.
19. Madhulika, Kabra SK, Barar V, et al. Upper and lower limb standards in newborn. *Indian pediatrics* 1989; **26**(7): 667-70.
20. Marchant T, Jaribu J, Penfold S, Tanner M, Armstrong Schellenberg J. Measuring newborn foot length to identify small babies in need of extra care: a cross sectional hospital based study with community follow-up in Tanzania. *BMC Public Health* 2010; **10**: 624.
21. Marchant T, Penfold S, Mkumbo E, et al. The reliability of a newborn foot length measurement tool used by community volunteers to identify low birth weight or premature babies born at home in southern Tanzania. *BMC Public Health* 2014; **14**: 859.
22. Mathur A, Tak SK, Kothari P. 'Foot length'--a newer approach in neonatal anthropometry. *Journal of tropical pediatrics* 1984; **30**(6): 333-6.
23. Merlob P, Sivan Y, Reisner SH. Lower limb standards in newborns. *American journal of diseases of children (1960)* 1984; **138**(2): 140-2.
24. Modibbo MH, Taura MG. Regression Equations for Birth Weight Estimation Using Anthropometric Measurements of Hand and Foot of Hausa New Born Babies in Kano-Nigeria. *Bayero Journal of Pure and Applied Sciences* 2013; **6**(1): 186-9.
25. Mukherjee S, Roy P, Mitra S, Samanta M, Chatterjee S. Measuring new born foot length to identify small babies in need of extra care: a cross-sectional hospital based study. *Iran J Pediatr* 2013; **23**(5): 508-12.

26. Nabiwemba E, Marchant T, Namazzi G, Kadobera D, Waiswa P. Identifying high-risk babies born in the community using foot length measurement at birth in Uganda. *Child: care, health and development* 2013a; **39**(1): 20-6.
27. Nabiwemba EL, Orach CG, Kolsteren P. Determining an anthropometric surrogate measure for identifying low birth weight babies in Uganda: a hospital-based cross sectional study. *BMC Pediatr* 2013b; **13**: 54.
28. Naqvi M, Valis D, Mohan P, Carroll A, Bickers P. Neonatal Foot Length - a Method of Gestational-Age Assessment (abstract only). *Pediatric research* 1986; **20**(4): A357.
29. Otupiri E, Wobil P, Nguah SB, Hindin MJ. Anthropometric measurements: options for identifying low birth weight newborns in Kumasi, Ghana. *PLoS One* 2014; **9**(9): e106712.
30. Paulsen CB, Nielsen BB, Msemo OA, et al. Anthropometric measurements can identify small for gestational age newborns: a cohort study in rural Tanzania. *BMC Pediatr* 2019; **19**(1): 120.
31. Pratinidhi AK, Bagade AC, Kakade SV, et al. Action-oriented colour-coded foot length calliper for primary healthcare workers as a proxy for birth weight & gestational period. *The Indian journal of medical research* 2017; **145**(3): 347-52.
32. Rakkappan I, Kuppusamy N. Newborn Foot Length Measurement to Identify High-risk Neonate. *International Journal of Scientific Study* 2016; **4**(2): 13-9.
33. Roy RA, Rao SS, Mithra P. Correlation of Foot Length and Gestational Maturity in Neonates-- A Study from Coastal Karnataka. *Indian J Public Health Research & Development* 2019; **10**(3): 231-5.
34. Rustagi N, Prasuna JG, Taneja DK. Anthropometric surrogates for screening of low birth weight newborns: a community-based study. *Asia Pac J Public Health* 2012; **24**(2): 343-51.
35. Sateesha SR, Niranjan HS, Jagadish AS, Benakappa N. Correlation of Foot & Hand Length Measurement with Gestational Maturity in Neonates *Int J Pharm Bio Sci* 2015; **6**(3): 1050-4.
36. Shah SS, Shrestha PS, Gami FC. Detection of Low Birth Weight Newborns by Foot Length as Proxy Measure of Birth Weight (abstract only). *Archives of disease in childhood* 2005; **90**(Suppl II): A9.
37. Singhal S, Tomar A, Masand R, Purohit A. A Simple Tool for Assessment of Gestational Age in Newborns using Foot Length. *Journal of Evolution of Medical and Dental Sciences* 2014; **3**(23): 6424-9.
38. Srinivasa S, Manasa G, Madhu GN. Foot length of newborn: Its correlation with gestational age and various anthropometric parameters. *Curr Pediatr Res* 2017; **21**(2): 248-53.
39. Srivastava A, Sharma U, Kumar S. To study correlation of foot length and gestational age of new born by new Ballard score. *Int J Res Med Sci* 2015; **3**(11): 3119-22.
40. Thawani R, Dewan P, Faridi MMA, Arora SK, Kumar R. Estimation of Gestational Age, Using Neonatal Anthropometry: A Cross-sectional Study in India. *Journal of Health Population and Nutrition* 2013; **31**(4): 523-30.
41. Thi HN, Khanh DK, Thu Hle T, Thomas EG, Lee KJ, Russell FM. Foot Length, Chest Circumference, and Mid Upper Arm Circumference Are Good Predictors of Low Birth Weight and Prematurity in Ethnic Minority Newborns in Vietnam: A Hospital-Based Observational Study. *PLoS One* 2015; **10**(11): e0142420.
42. Usher R, McLean F. Intrauterine growth of live-born Caucasian infants at sea level: standards obtained from measurements in 7 dimensions of infants born between 25 and 44 weeks of gestation. *J Pediatr* 1969; **74**(6): 901-10.
43. Vocel J, Markova H. [Signifivance of sole dermatoglyphics and of foot length in the accurate determination of gestational age in newborn infants]. *Cesk Pediatr* 1978; **33**(10): 618-20.
44. Hodgins S, Rajbhandari B, Joshi D, Ban B, Khatry SK, Mullany L. Community-Based Cluster Randomized Controlled Trial: Empowering Households to Identify and Provide Appropriate Care for Low-Birthweight Newborns in Nepal. *BMC Public Health* 2020; **Preprint**(https://www.researchsquare.com/article/rs-15575/v1).
